# Breast cancer metastasis to brain results in recruitment and activation of microglia through annexin-A1/formyl peptide receptor signaling

**DOI:** 10.1186/s13058-022-01514-2

**Published:** 2022-04-05

**Authors:** Sok Lin Foo, Karishma Sachaphibulkij, Corinne L. Y. Lee, Gracemary L. R. Yap, Jianzhou Cui, Thiruma Arumugam, Lina H. K. Lim

**Affiliations:** 1 Immunology Translational Research Program, Yong Loo Lin School of Medicine, National University of Singapore (NUS), 28 Medical Drive, Life Sciences Institute #03-06J, Singapore, 117456 Singapore; 2 Department of Physiology, Yong Loo Lin School of Medicine, National University of Singapore (NUS), Singapore, Singapore; 3 Immunology Program, Life Sciences Institute, National University of Singapore (NUS), Singapore, Singapore; 4 NUS Graduate School for Integrative Sciences and Engineering, NUS, National University of Singapore (NUS), Singapore, Singapore; 5 Present Address: Department of Physiology, Anatomy and Microbiology, School of Life Sciences, La Trobe University, Melbourne, Australia

**Keywords:** Annexin-A1, STAT3, FPR2, Microglia, Brain metastasis, Breast cancer

## Abstract

**Background:**

Despite advancements in therapies, brain metastasis in patients with triple negative subtype of breast cancer remains a therapeutic challenge. Activated microglia are often observed in close proximity to, or within, malignant tumor masses, suggesting a critical role that microglia play in brain tumor progression. Annexin-A1 (ANXA1), a glucocorticoid-regulated protein with immune-regulatory properties, has been implicated in the growth and metastasis of many cancers. Its role in breast cancer-microglia signaling crosstalk is not known.

**Methods:**

The importance of microglia proliferation and activation in breast cancer to brain metastasis was evaluated in MMTV-Wnt1 spontaneous mammary tumor mice and BALBc mice injected with 4T1 murine breast cancer cells into the carotid artery using flow cytometry. 4T1 induced-proliferation and migration of primary microglia and BV2 microglia cells were evaluated using 2D and coculture transwell assays. The requirement of ANXA1 in these functions was examined using a Crispr/Cas9 deletion mutant of ANXA1 in 4T1 breast cancer cells as well as BV2 microglia. Small molecule inhibition of the ANXA1 receptor FPR1 and FPR2 were also examined. The signaling pathways involved in these interactions were assessed using western blotting. The association between lymph node positive recurrence-free patient survival and distant metastasis-free patient survival and ANXA1 and FPR1 and FPR2 expression was examined using TCGA datasets.

**Results:**

Microglia activation is observed prior to brain metastasis in MMTV-Wnt1 mice with primary and secondary metastasis in the periphery. Metastatic 4T1 mammary cancer cells secrete ANXA1 to promote microglial migration, which in turn, enhances tumor cell migration. Silencing of ANXA1 in 4T1 cells by Crispr/Cas9 deletion, or using inhibitors of FPR1 or FPR2 inhibits microglia migration and leads to reduced activation of STAT3. Finally, elevated ANXA1, FPR1 and FPR2 is significantly associated with poor outcome in lymph node positive patients, particularly, for distant metastasis free patient survival.

**Conclusions:**

The present study uncovered a network encompassing autocrine/paracrine ANXA1 signaling between metastatic mammary cancer cells and microglia that drives microglial recruitment and activation. Inhibition of ANXA1 and/or its receptor may be therapeutically rewarding in the treatment of breast cancer and secondary metastasis to the brain.

**Supplementary Information:**

The online version contains supplementary material available at 10.1186/s13058-022-01514-2.

## Introduction

Metastasis is the greatest contributor to breast cancer mortality. While there is no existing cure for secondary or metastatic breast cancer, new treatments and combination of immunotherapies with chemotherapies are extending lives [1, 2]. This prolonged survival in breast cancer patients may also increase the frequency of breast cancer metastasis to the brain, a penultimate step prior to a loss in patient survival. Although genetic/epigenetic changes conveying chronic proliferative stimuli are essential to tumor progression, the larger impact of the tumor microenvironment (TME) on tumor progression is becoming increasingly evident [3]. Solid tumors can induce local and systemic immune aberrations to promote metastatic progression in distant organs, either by establishing a pre-metastatic niche, or by supporting colonization of distant target organs. In brain metastases, one of the prominent immune infiltrates are microglia, the resident parenchymal monocyte-lineage cells that function as a first line of defense during pathological insults in the central nervous system [4]. Originally, it was believed that the brain recruits microglia to the TME, to elicit an anti-tumor response. However, emerging evidence suggests that microglial recruitment to the brain TME generally favors metastatic progression and colonization of the brain, analogous to a wound, attracting immune cells to assist in healing. These pro-tumorigenic mechanisms typically involve activation of signal transducers and activators of transcription (STAT) 3, a DNA-binding transcription factor responsible for immune suppression and tumor evasion via transcriptomic changes in the immune cells [5]. Tumorigenic STAT3 activation has been frequently linked to more malignant cancer phenotypes, including growth, epithelial-mesenchymal transition, migration, invasion and metastasis [6]. Little is known, however, about the tumor-intrinsic factors secreted from breast cancer cells in transforming a distant metastatic site from a naive to a metastasis-promoting microenvironment following cancer cells infiltration into the brain. As such, the ability of tumor cells in inducing pro-tumorigenic phenotype in resident microglial cells might be determined by the distinct paracrine/juxtacrine mitogenic signals linked to the metastatic propensity and subtypes of breast cancer cells.

In this study, we link STAT3 with the induction and activation of an immunomodulatory protein, annexin-A1 (ANXA1). ANXA1 can be externalized and/or secreted to signal through two-cell surface receptors, formyl peptide receptor 1 (FPR1) and formyl peptide receptor 2 (FPR2), also known as ALXR in humans [7]. Besides being a pro-resolving mediator of inflammation, ANXA1 regulates a variety of remarkable salutary responses, including vesicle transport, signal transduction [8], cell transformation [9], cell matrix interaction and apoptosis [10], as well as endocrine and metabolic functions [11]. ANXA1 has been increasingly described to be highly expressed in metastatic basal-like breast cancer (BLBC) and triple-negative breast cancer (TNBC) [12–14]. Considering these multiple facets of ANXA1 in tumorigenesis and modulation of the immune system, it would be useful to delineate the roles of ANXA1 in the brain TME, aiding to inform strategies, such as prescription of the glucocorticoid dexamethasone, in managing symptoms related to elevated intracranial pressure and peritumoral edema in patients with brain metastasis [15, 16].

## Materials and methods

### Patient datasets

Kaplan–Meier survival curves for breast cancer were determined using https://kmplot.com/ [17]. Relapse-free survival (RFS) was available for 4929 patients, and out of these, 1656 patients were lymph node positive. Distant metastasis-free survival was available for 2765 patients, and out of these, 889 patients were lymph node positive. Data from all patients (treated or untreated) were used. All possible cut offs in the upper and lower quartiles are computed and the best performing threshold is used as the cut off.

### Animals

All animal work was approved by the NUS Institutional Animal Care and Use Committee (IACUC) and was in accordance with the National Advisory Committee for laboratory Animal Research (NACLAR) Guidelines (Guidelines on the Care and Use of Animals for Scientific Purposes). MMTV-Wnt transgenic mice were kind gift from Prof. David Virshup Laboratory (Duke NUS, Singapore), and the mice were backcrossed to C57BL/6 for maintenance. MMTV-Wnt+ mice usually develop mammary tumors between the age of 2 and 8 months. Mice were kept on a 12-h light/dark cycle with food and water provided ad libitum and maintained under pathogen-free conditions in the animal housing unit.

### MMTV-Wnt transgenic model of metastatic breast cancer with primary tumor resection

MMTV-Wnt transgenic mice were bred and monitored for development of palpable primary tumors approximately 1.5 cm in diameter. Surgery was performed for primary tumor resection, and the mice were allowed to recover. Upon development of relapse (2–3 weeks after primary tumor resection), the mice were euthanized and perfused with phosphate buffered saline (PBS). The brains were harvested for brain cell isolation.

### 4T1-Balb/c intracarotid injection brain metastasis model

BALB/c mice were anesthetized with isofluorane, and the right common carotid artery of the mice was carefully exposed and separated from vagal nerve. After tying one end loosely with a surgical ligature, one external carotid artery branch was tied tightly with another surgical ligature. By using a small syringe with a 32 G needle, 50 µL of stably transfected 4T1-luciferase cell suspension (10^4^ cells) was injected into the internal carotid artery. The loosely tied ligature on the common carotid artery was removed and the wound was sealed. Bioluminescence imaging was performed to monitor tumor seeding in the brain. Mice were euthanized, and the brains were harvested and isolated at the end of the study when significant weight loss or signs of distress was observed (Additional file 1: Methods).

### Isolation of immune cells from mouse brain

Mice were housed, bred and handled according to the Institutional Animal Care and Use Committee guidelines. Brains were isolated from brain cortices; meninges and choroid plexi were removed. Remaining brain tissue was mechanically dissociated in Dulbecco's phosphate-buffered saline (DPBS) (GE Healthcare Life Sciences, USA) using serological pipettes in (SPL Life Sciences). Cell suspension was filtered through 70 μm nylon mesh, centrifuged for 10 min at 400 r.c.f, 4 °C before being centrifuged over a 40% Percoll gradient (GE Healthcare Life Sciences, USA) for 20 min at 1000 r.c.f with minimal acceleration and deceleration, 4 °C.

### Flow cytometry

To characterize and quantify microglial cells, cells were stained with Fixable Viability Dye eFluor 506 (1:1000) (Thermo Fisher Scientific, USA) before incubated with Fc block (BioLegend, USA). Cells were stained with CD86-APC, CD45-APC/Cy7, CD11b-FITC, CD206-PE/Cy7, CD115-BV711 and CX3CR1-BV421 (BioLegend, USA). Cells were analyzed on a BD LSRFortessa (BD Biosciences, USA) or Attune NxT Flow Cytometer (Thermo Fisher Scientific, USA). Data analysis was performed using FlowJo software (FlowJo, USA). Microglia were identified as CX3CR1^+^CD11b^+^CD45^lo^ cells, and gated numbers and activation states were analyzed. Fluorescence minus one control (FMO) for each antibody was used to properly identify and gate the microglia population.

### Cell culture

All cells were grown in Dulbecco's Modified Eagle Medium: Nutrient Mixture F-12 (DMEM/F-12) Media (Biowest, France) supplemented with 10% heat-inactivated FBS (GE Healthcare Life Sciences, USA) and 1% penicillin–streptomycin (GE Healthcare Life Sciences, USA), in a humid, 37 °C atmosphere containing 5% CO_2_. Treatments with inhibitors Boc-MLF, 100 μM (Tocris, UK), WRW^4^, 10 μM (Tocris, UK) and UO126 10 μM [18] (InvivoGen, Hong Kong) were performed by adding them to the media for 24 h.

### Preparation of tumor cell conditioned media

4T1 and 4T07 murine mammary cancer cells were cultured in 10 mL of DMEM/F12 culture media supplemented with 10% heat-inactivated FBS, 1 mM l-glutamine, 1 mM sodium pyruvate, 100 U/mL penicillin and 100 μg/mL streptomycin in 75 cm^2^ cell culture flasks (Cellstar, Greiner Bio-one, Gloucestershire, UK) in a humidified incubator with 5% CO_2_ at 37 °C. The cells were allowed to reach 80–90% confluency before the media was removed and replaced with plain fresh media. Cell culture supernatant was harvested from adherent cancer cells after 24 h. The supernatant was centrifuged, filtered through a syringe filter membrane (0.2 µm) (Sartorius), and stored at − 80 °C until use as conditioned media (CM) with microglia cells.

### Primary mouse adult microglial culture

Brains were isolated from 2-to-6-month-old wild-type mice cortices, meninges and choroid plexi were removed. Remaining brain tissue was finely minced with a round-edge blade scalpel and dissociated. Cell suspension was filtered through 70-μm nylon mesh before 40% Percoll gradient separation (GE Healthcare Life Sciences, USA). Microglia and astrocyte co-cultures were maintained at 37 °C in 5% CO_2_ for 3–4 weeks, after which the confluent mixed glial cultures were subjected to mild trypsinization (0.05–0.12%) in the presence of 0.2–0.5-mM EDTA and 0.5–0.8-mM Ca^2+^. Isolated microglia were allowed to recover for 1 week and processed for cellular assays.

### Generation of genomic ANXA1 deletion cell lines using CRISPR-Cas9

Mouse ANXA1 CRISPR/Cas9 KO plasmid containing a pool of three plasmids each encoding the Cas9 nuclease and different gRNA plasmids 5′-TTTGATGCAGATGAACTCCG-3′, 5′-TCCATTCTCCTGTAAGTACG-3′ and 5′-GATCTGCTGGCGCTGAGCAT-3′ targeting ANXA1 were obtained from Santa Cruz Biotechnology, USA. Cells were transfected using Lipofectamine LTX and Plus Reagent (Invitrogen). For the establishment of ANXA1 knockout cell lines, mouse ANXA1 HDR Plasmid containing puromycin resistance gene was used for selection with puromycin (2 μg/mL) (Sigma-Aldrich, USA). Cells were then sorted for single RFP-positive cells in a 96-well plate with MoFlo XDP Cell Sorter (Beckman Coulter, USA). Single-cell colonies were then gradually expanded and analyzed by Western blotting for ANXA1 knockout clones.

### Cell proliferation assay

MTS assay was performed using CellTiter 96 AQueous One Solution Cell Proliferation Assay (Promega, Mannheim, Germany) as described by manufacturer. Cells were seeded into 96-well tissue culture plates at a density of 5 × 10^3^ cells per well and treated with CM or SFM control for 48–96 h. A tetrazolium compound (3-(4,5-dimethylthiazol-2-yl)-5-(3-carboxymethoxyphenyl)-2-(4-sulfophenyl)-2*H*-tetrazolium, inner salt; MTS) containing solution was added into each well and incubated for 2 h. Absorbance at 490 nm was measured using Spark microplate reader (Tecan, Männedorf, Switzerland).

### In vitro migration assay

Migration of BV-2 microglial cells was evaluated using a chemotaxis Boyden chamber system with 24-well insert, with 8.0 μm pore size polycarbonate membrane separating upper and lower wells (SPL Life Sciences, Korea). 1 × 10^5^ cells/100 μl/well were seeded into the upper inserts, while treatment media was applied to the lower wells and incubated at 37 °C in 5% CO_2_. Boc-MLF and WRW4 were purchased from Tocris, UK. After 24 or 48 h, the migrated cells attached to the bottom of the insert were stained with crystal violet for quantification. The number of cells migrated were acquired in five randomized fields using SZX16 stereomicroscope (Olympus, USA). Numbers of migrated cells were quantified using ImageJ software (NIH, USA).

### Western blot analysis

Protein concentration was determined by Bradford’s protein assay (BioRad Laboratories, USA). Proteins were separated by 8–12% sodium dodecyl sulfate–polyacrylamide gel electrophoresis (SDS-PAGE) and transferred to a polyvinylidene difluoride (PVDF) membrane (BioRad Laboratories, USA). Membranes were incubated with antibodies against Annexin A1 (1:1000), p44/42 MAPK (ERK1/2) (1:1000), Phospho-p44/42MAPK (ERK1/2) (Thr202/Tyr204) (1:1000), SAPK/JNK (1:1000), Phospho-SAPK/JNK (Thr183/Tyr185) (1:1000), Stat1 (1:1000), Phospho-Stat1 (Tyr701) (1:1000), Stat3 (1:1000), Phospho-Stat3 (Tyr705) (1:1000), Actin (1:5000) and GAPDH (14C10) (1:1000) (Cell Signaling, Boston, USA) and horseradish peroxidase (HRP)-conjugated secondary antibodies (1:10,000, Thermo Fisher Scientific, USA). Proteins were visualized by chemiluminescence detection reagent (GE Healthcare Europe, Portugal) using ChemiDoc XRS + System (Bio-Rad, USA). β-actin or GAPDH was used as internal loading controls.

### Immunoprecipitation

Cells were harvested and lysed in lysis buffer IP. After centrifugation of cells at 4 °C, protein was quantified using 1× Bradford’s reagent (BioRad Laboratories, USA) and BSA standards (Thermo Scientific). 20 μL of Protein A/G Plus-Agarose beads (Santa Cruz Biotechnology, USA) were washed and added to each lysates for pre-clearing. After incubation of 50 μL of beads with control IgG or specific antibody for 4 h to form antibody-coupled beads, the pre-cleared lysates were mixed and incubated for overnight. Following day, 4× loading buffer was added to beads after washing the beads. The antibody-coupled beads were heated at 100 °C for 10 min before proceeding to Western blot analysis.

### Immunocytochemistry

Primary adult microglial cells on glass cover slips fixed with 4% PFA were double-labelled for ANXA1 and pan-marker of microglia cell-type, Iba1. Cells were blocked with blocking buffer for 1 h at RT before incubation with rabbit anti-ANXA1 (1:200, Cell Signaling Technology, USA) and goat anti-Iba1 (1:200, Novus Biologicals, USA) overnight at 4 °C. Alexa Fluor® 568 anti-goat secondary antibodies and Alexa Fluor® 488 anti-rabbit secondary antibodies (1:500, Invitrogen, USA) were applied for 1 h at RT. DAPI DNA counterstain (1:5000) AbD Serotec, UK) was added to the cells for 10 min. Cells were washed with washing buffer before mounting with Vectashield Fluorescent Mounting Medium (Vector Laboratories, USA). The coverslip edges were sealed and stored in dark 4 °C until imaging with LSM 510 confocal (Carl Zeiss, Germany).

### RNA isolation, cDNA synthesis and quantitative real-time PCR

Total RNA was extracted from the cells by using Direct-zol RNA Miniprep Plus Kit (Zymo Research, USA). Total RNA and purity of RNA were quantified and determined using NanoDrop 1000 spectrophotometer (Thermo Fisher Scientific, Massachusetts, USA). Desired amount of total RNA was converted into first-strand cDNA GoScriptTM Reverse Transcriptase System (Promega) according to manufacturer’s protocol. cDNA was amplified on a thermal cycler (BioRad Laboratories, USA) with annealing step at 25 °C for 5 min, extending step at 42 °C for up to 1 h and the reaction was stopped at by inactivating the reverse transcriptase at 70 °C for 15 min. Synthesized cDNA was mixed to the specific forward and reverse primers (Additional file 1: Table S1) and Luna Universal qPCR Master Mix (NEB, USA). Real-time polymerase chain reaction (RT-PCR) was initiated using Applied Biosystems 7500 Real-Time PCR System (Thermo Fisher Scientific, Massachusetts, USA). The amplification program was 95 °C for 5 min followed by 40 cycles of 95 °C for 10 s, 60 °C for 10 s and 72 °C for 10 s. Reactions were run in duplicate in at least three independent experiments. The results were normalized to the expression of GAPDH. Gene expression was analyzed using 2^−ΔCT^ and 2^−ΔΔCT^ methods [19].

### Statistical analysis

Data are expressed as means and error bars represent standard error of mean (SEM) from three independent experiments. Unpaired two-tailed Student’s *t* test was used for comparison of one variable between two groups. One-way analysis of variance (ANOVA) followed by Tukey’s or Dunnet’s multiple comparison post hoc test was used to determine inter-group differences between more than two groups for one variable. Two-way ANOVA followed by Sidak’s multiple comparison post hoc test was used to determine inter-group differences between more than two groups for two variables. The level of statistical significance was taken at *P* < 0.05 throughout the study.

## Results

### Activation of CD11b^+^CD45^lo^ brain resident microglial cells in primary and secondary mammary tumors-bearing MMTV-Wnt1 mice

**Fig. 1:**
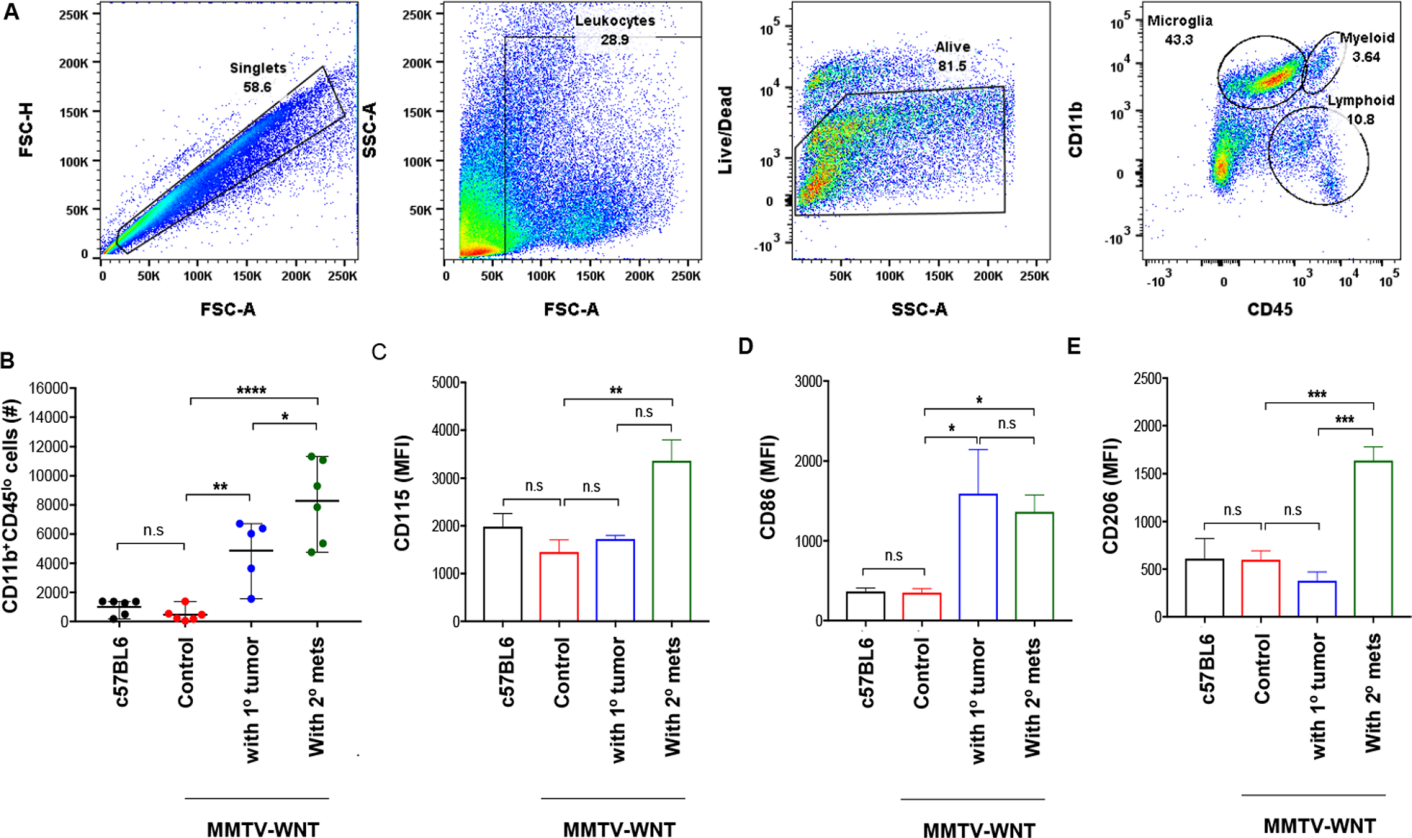
Activation of CD11b^+^CD45^lo^ brain resident microglial cells in primary and secondary mammary tumors-bearing MMTV-Wnt1 mice. **a** Representative FACS showing microglia from the brains of mice bearing primary tumor or MMTV-Wnt1 mice bearing secondary tumors. **b** Flow cytometry analysis of number of microglial cells in the brains of indicated mice. **c** CD115, **d** CD86 pro-inflammatory marker, and **e** CD206 anti-inflammatory marker expression on microglial cells from tumor mice. MFI, median fluorescence intensity. Data represents mean ± standard error of mean (SEM) of *n* = 5–6 mice per group. *P < 0.05, **P < 0.01, ***P < 0.001, ****P < 0.0001

We first analyzed metastasis to the brain in spontaneous mammary cancer MMTV-Wnt1 mice with either primary or secondary mammary tumors. Tumors were resected when they developed, which allowed the residual and circulating cells to grow again and metastasize. Three-week post-primary tumor resection, systemic metastases were observed predominantly in the lungs, lymph nodes and liver upon necropsy. However, neither microscopic analysis of histopathology staining nor clonogenic assays demonstrated evidence of metastasis in the brain (Additional file 1: Fig. S1, A and B). Flow cytometry analysis of microglial cells from these brains, analyzed after sequential gating (Fig. 1a), however, revealed an increase in the number of CD11b^+^CD45^lo^ microglial cells gated in secondary metastatic tumor-bearing MMTV-Wnt1 mice, compared to non-tumor-bearing and primary tumor-bearing MMTV-Wnt1 mice (Fig. 1b). The increase in CD11b^+^CD45^lo^ microglia was observed with enhanced cell surface expression of CD115 (the colony stimulating factor 1 receptor, CSF1R) on microglia from the brains of secondary tumor-bearing MMTV-Wnt1 mice (Fig. 1c). CD86, a pro-inflammatory marker, was upregulated in both MMTV-Wnt1 mice bearing primary and secondary mammary tumors (Fig. 1d). However, upregulation of CD206, an anti-inflammatory cell surface marker, was solely observed in secondary tumor-bearing MMTV-Wnt1 mice, and not primary tumor or non-tumor bearing mice (Fig. 1e), indicating that potential soluble factors secreted by secondary tumors may activate microglia and promote anti-inflammatory/pro-tumorigenic properties.

### Secretome from 4T1 metastatic mammary cancer cells promotes microglial proliferation, directional migration and activation

**Fig. 2:**
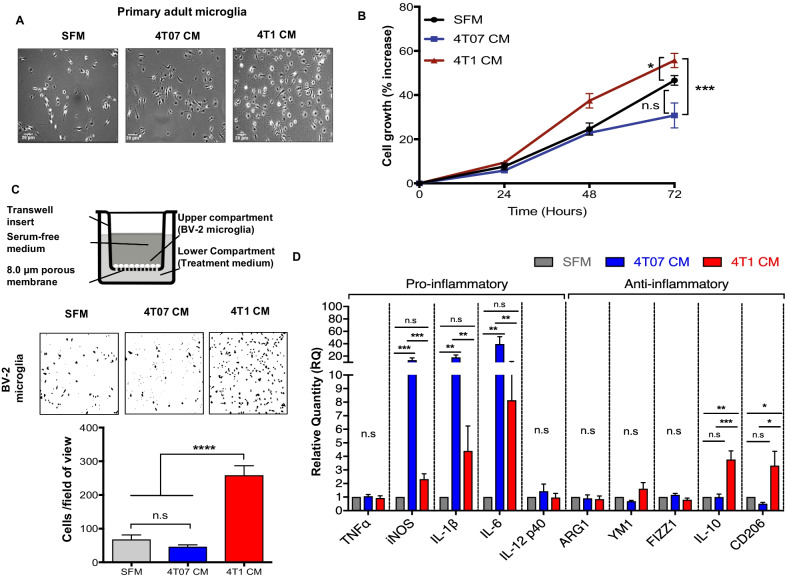
Secretome from 4T1 metastatic mammary cancer cells promotes microglial proliferation, directional migration, and activation. **a** Representative brightfield images of primary adult mouse microglia. **b** Growth curves of BV-2 microglial cells upon exposure to SFM, 4T07 CM and 4T1 CM analyzed using MTT assay. **c** Microglial cells were plated in the upper compartment of a boyden chamber chemotaxis assay, with either SFM, 4T07 CM or 4T1 CM the as chemoattractant for 24 h. Representative brightfield images of migrated BV-2 microglial cells captured at 100× magnification and quantification of migrated cells after 24 h. **d** BV-2 microglia were treated with either SFM, 4T07 CM or 4T1 CM and gene expression of inflammatory markers was determined by qRT-PCR. Data represent mean ± SEM; *n* = 3 independent experiments. Brightfield images captured at 100× magnification, representative of three independent experiments. *P < 0.05, **P < 0.01, ***P < 0.001, ****P < 0.0001

To investigate the role of these potential soluble factors secreted by metastatic mammary tumor cells, we compared 4T1 (metastatic) and 4T07 (invasive but non-metastatic) cell lines. Using these cell lines, we investigated whether soluble factors secreted by either metastatic or non-metastatic mammary cancer cells, can regulate microglial proliferation, migration and gene transcription of pro- and anti-inflammatory markers in vitro. Conditioned media (CM) was collected from 4T1 and 4T07 cells and used to stimulate microglial cells. Exposure of 4T1 CM on primary adult mouse microglia, induced marked growth-stimulating effects at 24 h, where an increase in the density of primary microglial cells incubated with 4T1 CM was observed, but not with 4T07 CM or serum-free media (SFM) control (Fig. 2a). Similar observations were also obtained in murine BV-2 microglial cells, where 4T1 CM, but not 4T07 CM or SFM control, induced microglia growth stimulation over 72 h of treatment (Fig. 2b).

To determine if the soluble factors present in the secretome of either 4T1 or 4T07 cancer cells influence the migratory properties of microglial cells, transwell migration was performed and results obtained showed that 4T1 CM induced significant transmigration of BV-2 microglia compared to SFM and 4T07 CM (Fig. 2c). Furthermore, 24-h incubation with 4T07 CM significantly upregulated inflammatory gene expression for three out of the five pro-inflammatory markers assessed (iNOS, IL-1β, and IL-6), but not the anti-inflammatory genes (Fig. 2d). In addition, exposure to 4T1 CM induced gene expression of the anti-inflammatory genes IL-10 and CD206, but not the pro-inflammatory genes in BV-2 microglia (Fig. 2d). These results suggest that the increased proliferation and migration abilities observed in BV-2 microglial cells inoculated with metastatic 4T1 CM, may possibly be attributed to promote a more immunosuppressive/pro-tumorigenic phenotype observed in these cells.

### ANXA1 in 4T1 metastatic mammary cancer cells secretome promotes directional migration of BV-2 microglia

Functional analysis of 4T1 CM and 4T07 CM on microglia, suggested that microglial cells are influenced by paracrine factors expressed and secreted by cancer cells. ANXA1 has been previously shown by our laboratory [13, 20, 21], to be a potential marker for discrimination of TNBC- or basal-like breast cancers (BLBC) from other breast cancer subtypes [12]. Therefore, we investigated differential expression of ANXA1 in 4T1 metastatic and 4T07 non-metastatic mammary cancer cells. As expected, ANXA1 expression was strikingly higher in 4T1 cells compared to 4T07 cells, at both the RNA and protein levels (Additional file 1: Fig. S2, A and B). As ANXA1 has been reported to be found in the extracellular space or bound to the outer leaflet of the plasma membrane [22], ANXA1 externalized by 4T1 cells were also detected in the CM of 4T1 cells (Additional file 1: Fig. S2C).

**Fig. 3:**
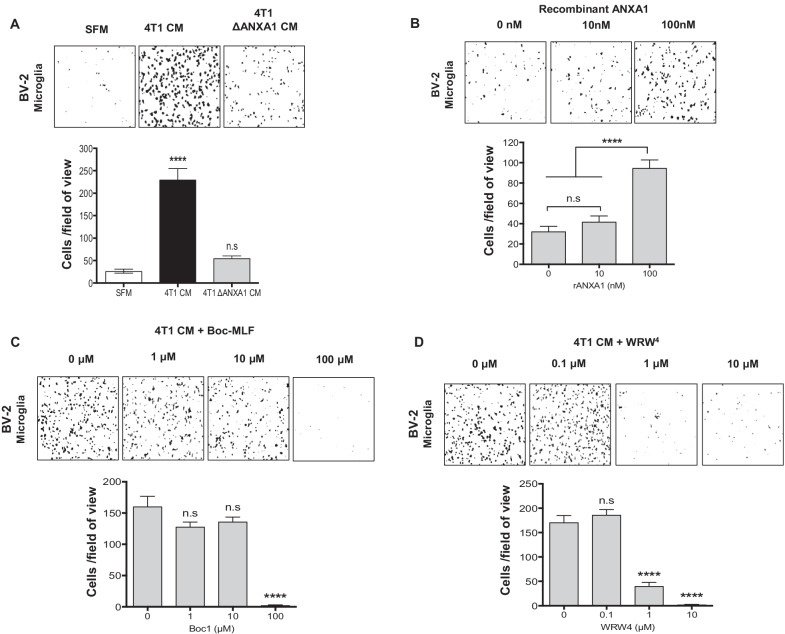
ANXA1 in 4T1 metastatic mammary cancer cells secretome promotes BV-2 microglial directional migration. **a**–**d** BV-2 microglial cells were treated with indicated concentrations of **a** SFM, 4T1 CM or 4T1 ΔANXA1 CM, **b** recombinant ANXA1 protein, **c** 4T1 CM supplemented with Boc-MLF FPR1 antagonist or **d** WRW4 FPR2 antagonist for 24 h in transwell migration assay. Data represent mean ± SEM; *n* = 3 independent experiments. Images are representative of three independent experiments. ****P < 0.0001

Next, to further investigate the contribution of secreted ANXA1 on BV-2 microglia, ANXA1 was depleted by generating a CRISPR-Cas9 ANXA1 knock-out 4T1 cell line (ΔANXA1 4T1) (Additional file 1: Methods and Additional file 1: Fig. S3). Analyses of CM from ΔANXA1 4T1 revealed that ANXA1 was not detected in ΔANXA1 4T1 CM (Additional file 1: Fig. S4A). The proliferation of BV-2 microglia upon exposure to ΔANXA1 4T1 CM was not significantly different compared to BV-2 microglia with 4T1 CM (Additional file 1: Fig. S4B). We verified the presence of cell surface expression of the receptors for ANXA1, FPR1 and FPR2, the G protein coupled receptors, by qRT-PCR and western blot analysis (Additional file 1: Fig. S4, C and D), and furthermore, results obtained revealed no significant difference in proliferation when BV-2 microglia was treated with increasing concentrations of either FPR1 (Boc-MLF) or FPR2 (WRW4) inhibitors (Additional file 1: Fig. S4, E and F). These data suggest that 4T1-derived ANXA1 was not responsible for the growth promoting effect of 4T1 CM on microglia. However, depletion of ANXA1 in 4T1, inhibited directional migration of BV2, indicating that 4T1-derived ANXA1 was involved in microglia migration (Fig. 3a). To further support these findings, we also showed that exogenous recombinant ANXA1 protein induced the migration of BV-2 microglial cells in an concentration-dependent manner (Fig. 3b). Furthermore, addition of FPR1 or FPR2 antagonists (Boc-MLF or WRW4) in 4T1 CM, dose-dependently inhibited the directional chemotaxis induced by 4T1 CM on BV-2 microglial cells (Fig. 3c, d), indicating that activation of the ANXA1 receptors, can enhance the migration of BV-2 microglia cells.

### Tumor-expressed ANXA1 increases the seeding, but not growth of 4T1 tumors in the brain

**Fig. 4:**
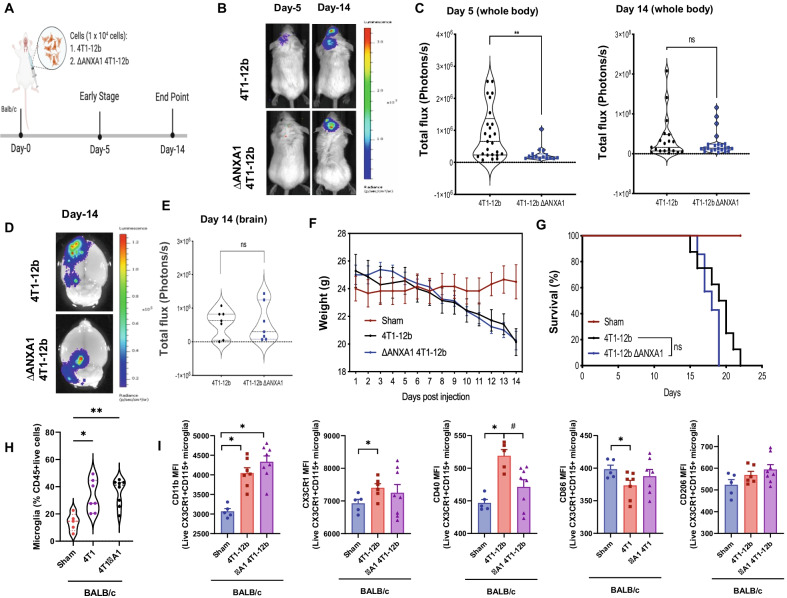
ANXA1 expressed in 4T1 cells injected into the carotid artery may enhance initial tumor growth. **a** Diagram depicting model and cells injected into BALB/c mice. **b** Mice were injected with 4T1-luminescent cells into the intracarotid artery. Representative bioluminescence images and **c** violin plot quantification of brain metastases at Day-5 and Day-14 after intracarotid injection of 4T1-12b cells. **d**, **e** Representative bioluminescence images of the brains and violin plot quantification of brain metastases 14 days after intracarotid injection of 4T1-12B cells. **f** Weight loss and **g** Survival rates after injection of parental 4T1 or the ΔANXA1-4T1 tumor cells. **h** The ratio of CD45lo/CD11b+ Microglia in the brain at Day 14 analyzed by flow cytometry. **i** Expression of CD11b, CX3CR1, CD40, CD86 and CD206 in microglia. *P < 0.05, **P < 0.01, versus Sham, ^#^*P* < 0.05 versus 4T1

To assess the effect of tumor intrinsic ANXA1 in an in vivo system, we utilized a model of brain metastasis via intra-carotid injection of 4T1-12B (luciferase expressing) cells. We compared the parental 4T1 and ΔANXA1 4T1 to examine the effect of tumor intrinsic ANXA1 in brain metastasis (Fig. 4a). Intracarotid artery injection of 4T1 cells into ANXA1^+/+^ mice resulted in significant luminescence in the brain, at Day 5 and Day 14 post-injection (Fig. 4b–d) on the ipsilateral side of the brain, and dramatic weight loss and significantly reduced survival (Fig. 4e, g).

In addition, a slight delay in tumor infiltration into the brain at day 5 can be observed when ΔANXA1 4T1 cells were injected (Fig. 4b, c), but at day 14, no difference in tumor growth was seen in mice injected with parental 4T1 or the ΔANXA1-4T1 tumor cells (Fig. 4d). Furthermore, there was no difference in tumor burden in the brains of both groups of mice injected with either the parental 4T1 or the ΔANXA1 4T1 cells (Fig. 4f–g). Injection of 4T1 cells, regardless of the expression of ANXA1, caused weight loss and a significant reduction in overall survival of the mice, suggesting that expression of ANXA1 in 4T1 cells did not significantly affect the tumor growth in the brain, supporting our in vitro observation.

We next determined if any changes in leukocyte populations in the brain occurs in mice with brain tumors, using multicolor flow cytometry. Injection of ΔANXA1-4T1-12B tumor cells into mice did not significantly alter the recruitment of microglia into the brain at 14 days, when compared to 4T1 cells (Fig. 4h). Expression of CD11b and CXC3CR1 in the microglia were all increased with 4T1 injection, irrespective of ANXA1 expression in the tumors (Fig. 4i). Interestingly, CD40 expression was significantly increased in microglia in mice injected with parental 4T1-12B cells and reduced with ΔANXA1-4T1-12B. The pro-inflammatory marker CD86 was reduced with 4T1 and not for ΔANXA1-4T1-12B with no change in the anti-inflammatory marker CD206. This data indicates that tumor expressed ANXA1 may not be important for number of microglia yet may be important for the activation of microglia in the brain upon brain metastases.

### FPRs antagonism attenuated gene expression of IL-6 and IL-10 induced by 4T1 metastatic mammary cancer cells conditioned media

**Fig. 5:**
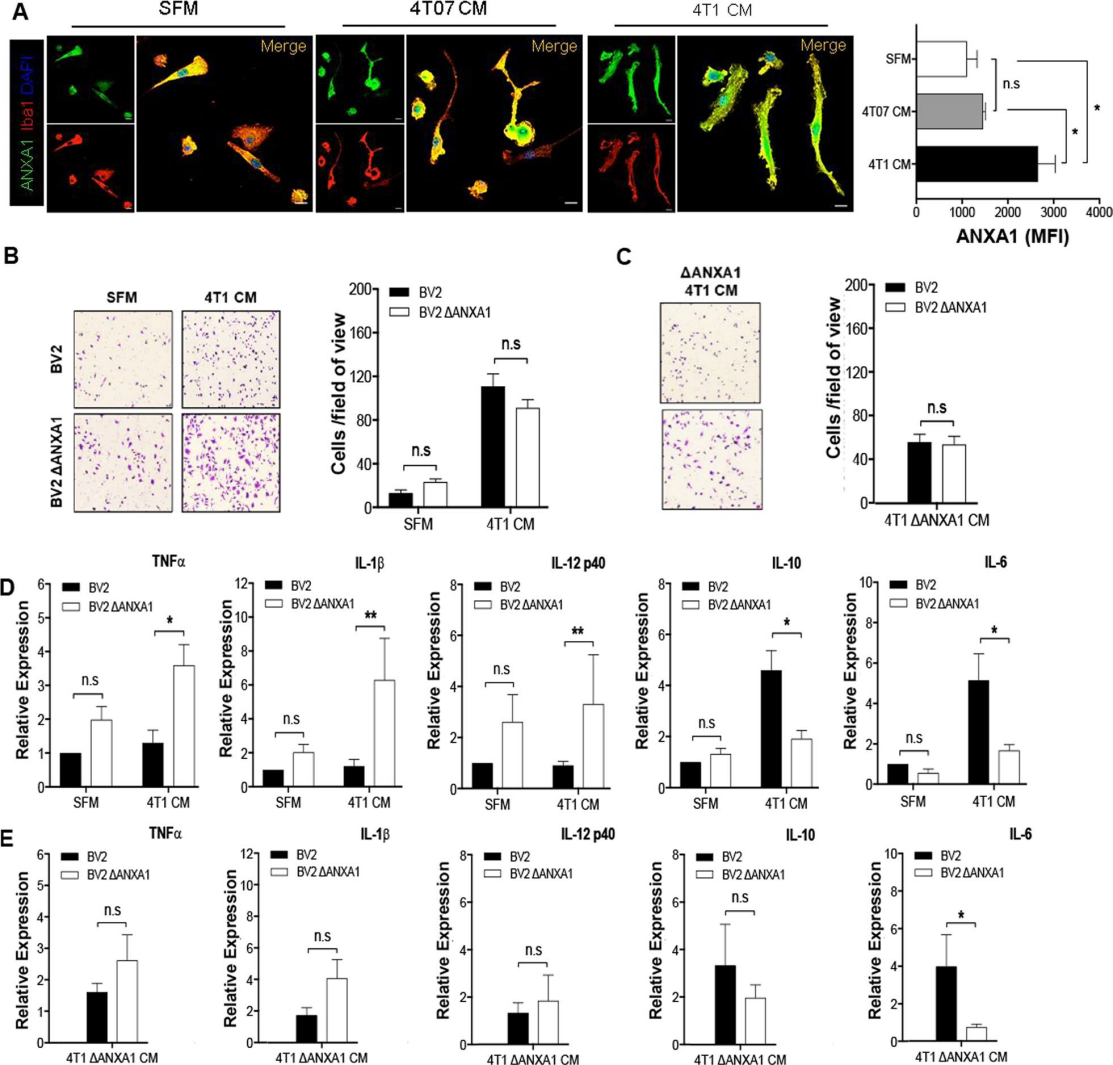
Extracellular and intracellular ANXA1 elicited different effects on migratory profiles in BV-2 microglial cells. **a** Primary adult microglia treated with either SFM, 4T07 CM, or 4T1 CM for 24 h and stained with ANXA1 (green), microglial marker Iba1 (red), and DNA-binding dye DAPI (blue). Graph (rightmost) showing the immunoreactivity against ANXA1 on primary adult microglial cells. **b** and **c** BV-2 or BV-2 ΔANXA1 microglia were treated with SFM, 4T1 CM or 4T1 ΔANXA1 CM for 24 h in the transwell migration assay. Representative brightfield images and quantification of migrated BV-2 or BV-2 ΔANXA1 microglia. **c** BV-2 or BV-2 ΔANXA1 microglia were treated with SFM, 4T1 CM or 4T1 ΔANXA1 CM for 24 h and gene expression of inflammatory markers were analyzed using qRT-PCR. Data represent mean ± SEM; *n* = 3 independent experiments. *P < 0.05, **P < 0.01

To determine if FPR signaling is involved in inflammatory gene expression in microglia treated with 4T1 CM, Boc-MLF (FPR1 antagonist) or WRW4 (FPR2 antagonist) was added. Only IL-6 was significantly down-regulated with the addition of either to 4T1 CM on BV-2 microglia, compared to with 4T1 CM alone, with no effect on the other pro-inflammatory genes measured (TNFα, IL-1β, IL-12p40), while expression of three anti-inflammatory markers, ARG1, IL-10 and CD206 were significantly down-regulated upon treatments with 4T1 CM and either FPR1 or FPR2 antagonists (Additional file 1: Fig. S5). These results suggest that FPR signaling enhances the immunosuppressive/tumor promoting phenotype of microglial cells induced by 4T1 CM, independent of ANXA1 expression in 4T1 CM. Therefore, we postulated that ANXA1 may be secreted by microglia in this system. Using a combination of immunostaining and western blots analyses, we demonstrated that ANXA1 could be detected in adult mouse brain, co-localized with the immunoreactivity of microglial marker CX3CR1, and both the primary and BV-2 microglial cells expressed ANXA1 basally (Additional file 1: Fig. S6, A and B, Fig. 5a). A significant up-regulation of ANXA1 expression was observed in primary and BV-2 microglial cells post-24-h treatment with 4T1 CM and not 4T07 CM (Additional file 1: Fig. S6, C and E). Thus, in line with our hypothesis, ANXA1 was detected in the supernatant of BV-2 microglia incubated with ΔANXA1 4T1 CM.

### Intracellular ANXA1 inhibits pro-inflammatory markers in BV-2 microglial cells

To determine whether the endogenously expressed ANXA1 in microglia (autocrine) plays a role in microglia activation and recruitment in response to cancer, ANXA1 was deleted in BV-2 microglia cells using CRISPR-Cas9 (ΔANXA1 BV-2) (Additional file 1: Methods and Additional file 1: Fig. S7), and transwell migration and gene expression profiles of pro- and anti-inflammatory markers were assessed in parental and ΔANXA1 BV-2 microglial cells. Migration of ΔANXA1 BV-2 microglial cells induced by 4T1 CM was not significantly different from parental BV-2 microglial cells (Fig. 5b). These results suggest that the migration of BV-2 microglial cells was induced by exogenous ANXA1 present in the 4T1 CM, and not regulated by endogenous ANXA1 within microglia. To confirm this, migration was assessed with CM from ΔANXA1-4T1 cells in BV2 and ΔANXA1 BV-2 microglia. BV2 microglia migrated less with ΔANXA1-4T1 CM as compared to 4T1 CM, and this was not significantly different in ΔANXA1-BV2 microglia (Fig. 5c). This indicates that microglia migration is indeed dependent on exogenous ANXA1 secreted from cancer cells.

We next examined the levels of inflammatory/anti-inflammatory markers in BV2 microglia cells and ΔANXA1 BV-2 microglia upon exposure to 4T1 CM. Gene expression of pro-inflammatory TNFα, IL-1β and IL-12p40 were significantly higher (Fig. 5d) while IL-6 and IL-10 were significantly down-regulated after incubation with 4T1 CM in ΔANXA1 BV-2 microglia compared to parental BV-2 microglia. In addition, ΔANXA1 BV-2 microglia expressed higher levels of FPR1 but not FPR2 basally and in the presence of 4T1 CM (Additional file 1: Fig. S8). To determine if exogenous ANXA1 is important in the anti-inflammatory phenotype observed in microglia, CM from ΔANXA1-4T1 cells was added to BV2 and ΔANXA1 BV-2 microglia. As shown in Fig. 5e, TNFα, IL-1β and IL-12p40 showed similar trends of upregulation, while IL-6 and IL-10 were downregulated in ΔANXA1 BV-2 microglia treated with ΔANXA1-4T1 CM, not significantly different from ΔANXA1 BV-2 microglia treated with 4T1 CM. The larger cell soma, as well as the enhanced expressions of TNFα, IL-1β and IL-12p40 pro-inflammatory markers in ΔANXA1 BV-2 microglia incubated with 4T1 CM, suggest that expression of ANXA1 in microglia promotes an anti-inflammatory/pro-tumorigenic phenotype.

In all, these data indicate that ANXA1 secreted from 4T1 cells drives microglial migration, while ANXA1 expressed in microglia is important for microglia activation and cytokine expression.

### Exogenous ANXA1 inhibits STAT3 signaling through FPRs in microglia

We next explored the signaling pathways which could be involved in the ANXA1-dependent crosstalk between metastatic cancer cells and microglial cells. The observations that the genes for M1 pro-inflammatory cytokine IL-6 and M2 anti-inflammatory cytokine IL-10 emerged to be induced following exposure of BV-2 microglial cells to 4T1 CM, and the consistent inhibition of the cytokines by FPRs antagonists Boc-MLF and WRW4, provided rationale to explore the transcription factors regulating these two cytokines (Additional file 1: Fig. S9A). Using CytReg database (https://cytreg.bu.edu/search.html), we identified signal transducer and activator of transcription (STAT) STAT1 and STAT3 among the transcription factors regulating gene expression of both IL-6 and IL-10. STAT1 and STAT3 were selected, as these two transcription factors are known to play prominent roles in tumorigenesis and tumor-associated immunosuppression [23, 24]. In addition, ANXA1 has been shown to be released from apoptotic cells and dampen inflammation through STAT3 [25], and FPR2 is regulated by STAT3 [26]. Based on the gene regulatory network interaction from Cytreg database, STAT1 has a repressing interaction on IL-6 transcription, while STAT3 results in transcriptional activation of both IL-6 and IL-10 (Additional file 1: Fig. S9B).

**Fig. 6:**
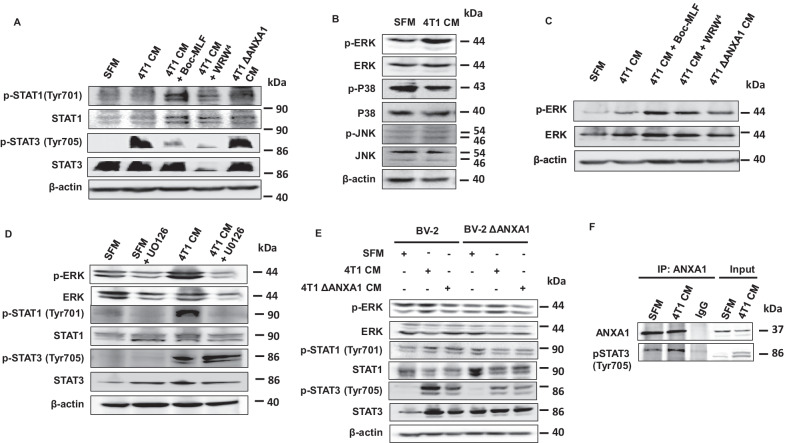
ANXA1 regulates STAT3 signaling through FPRs in microglia. **a**, **c** and **d** BV-2 microglia were treated with SFM, 4T1 CM, 4T1 ΔANXA1 CM or 4T1CM with the indicted inhibitors for 24 h. **b** BV-2 microglia were treated with SFM or 4T1 CM for 24 h. **c** BV-2 or BV-2 ΔANXA1 microglial cells were treated with SFM, 4T1 CM or 4T1 ΔANXA1 CM for 24 h. Indicated proteins were analyzed using western-blot analysis. **f** BV-2 microglia were treated with SFM or 4T1 CM for 24 h. The interaction between ANXA1 and phosphorylated STAT3 was assessed using immunoprecipitation (IP). Images shown are representative of at least 3 independent biological replicates. Loading control: β-actin

Pharmacological inhibition of either FPR1 or FPR2 enhanced the activation of STAT1, with a greater activation observed with FPR1 inhibition (Fig. 6a). On the contrary, activation of STAT3 was reduced with pharmacological inhibition of FPR1 and more so FPR2 (Fig. 6a). Phosphorylation of both STAT1 and STAT3 was also observed in BV-2 microglia treated with ΔANXA1 4T1 CM (Fig. 6a). The result aligned with the idea that ANXA1 could be an autocrine factor secreted by BV-2 microglia, which was sufficient to activate FPR1/2, as well as the downstream activation of STAT3. Next, as STATs may be regulated by MAP kinase activation, we determined if ERK1/2, p38 or JNK was activated after 4T1 CM treatment. Phosphorylation of ERK1/2, but not p38 or JNK was increased at 24-h post-exposure to 4T1 CM (Fig. 6b). Surprisingly, concomitant exposure of BV-2 microglia with 4T1 CM and either FPR1 antagonist, Boc-MLF or FPR2 antagonist, WRW4, further enhanced the activation of ERK1/2 (Fig. 6c). BV-2 microglia subjected to ΔANXA1 4T1 CM also resulted in enhanced phosphorylation of ERK1/2, compared to parental 4T1 CM (Fig. 6c), suggesting that ANXA1 and FPR1/2 agonists, inhibit the activation of ERK1/2 following 4T1 CM.

We next determined if inhibition of ERK1/2 activation would modulate the activation of STAT3. Treatment of BV-2 microglial cells for 24 h with 4T1 CM in the presence of 10 μM U0126 (a highly selective inhibitor of MEK1/2), resulted in decreased phosphorylation of ERK1/2 (Fig. 6d). Consistent with our hypothesis, inhibition of ERK1/2 activation notably enhanced the activation of STAT3 compared to treatment with 4T1 CM without UO126, demonstrated by the elevated phosphorylation of STAT3 at Tyr705 (Fig. 6d). In contrast, U0126 abolished the activation and phosphorylation of STAT1 ^Tyr701^ induced by 4T1 CM (Fig. 6d). These findings provide evidence that FPR activation activates STAT3 to antagonize the ERK1/2-STAT1 axis in microglial cells exposed to 4T1 CM.

### Endogenous ANXA1 enhances STAT3 activation in microglia

We next investigated whether endogenous ANXA1expressed in microglia played a functional role downstream of FPRs activation. ERK1/2, p38, and JNK activation was determined in parental and ΔANXA1 BV-2 microglial cells treated with 4T1 CM. Enhanced phosphorylation of ERK1/2 was consistently detected in both the parental and ΔANXA1 BV-2 microglial cells exposed to 4T1 CM (Fig. 6e). There was no discernable difference in p38, JNK or ERK activation between WT and ΔANXA1 BV-2 microglial (Fig. 6e), indicating that exogenous ANXA1-FPRs signaling was not affected by ANXA1 deletion in the microglia (Additional file 1: Fig. S10). ΔANXA1 BV-2 microglia treated with 4T1 CM and ΔANXA1 4T1 CM expressed lower levels of phospho-STAT3 compared to WT BV-2 microglia (Fig. 6e). The attenuated STAT3 activation induced by 4T1 CM in ΔANXA1 BV-2 microglia, despite similar degree of ERK1/2 phosphorylation downstream of FPRs activation, revealed that endogenous ANXA1 could act on the same pathway by affecting the phosphorylation of STAT3 to synergistically enhance its activation. The absence of ANXA1 in BV-2 microglia led to a prominent reduction in STAT3 activation following 4T1 CM treatment compared to WT BV-2 microglia. Co-immunoprecipitation assays confirmed intracellularly expressed ANXA1 in BV-2 microglia formed a complex with phosphorylated STAT3 upon 4T1 CM treatment (Fig. 6f). It is plausible that this interaction could either enhance the phosphorylation of STAT3, or prevent the dephosphorylation of phosphor-STAT3, thereby enhancing, or maintaining, the activation of STAT3.

### Expression of ANXA1/FPR2 in breast cancer (BRCA) TCGA cohort and distant metastasis survival

**Fig. 7:**
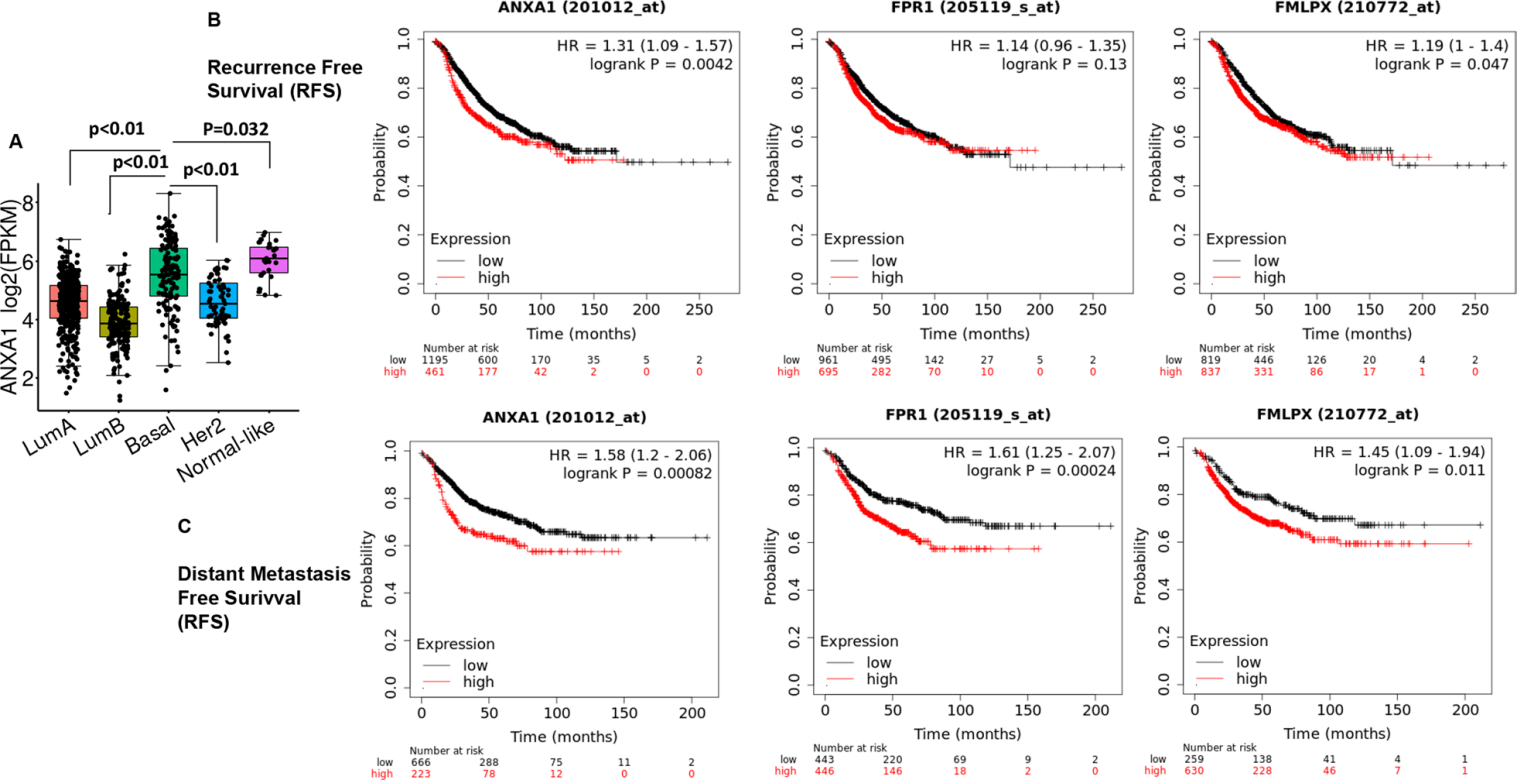
Expression and metastatic prognosis of ANXA1, FPR1 and FPR2 in clinical breast cancer samples. **a** The gene expression pattern analysis of ANXA1 in the various subtypes of breast cancers **b** Kaplan–Meier survival curve analysis for recurrence-free survival (RFS) for lymph node positive breast cancer patients **c** Kaplan–Meier survival curve analysis for distant metastasis free survival (DMFS) for lymph node positive breast cancer patients

Finally, the overall expression level changes in ANXA1 in the various subtypes of breast cancer and brain cancer using was determined using TCGA (Fig. 7a). Expression of ANXA1 is significantly higher in basal and normal-like breast cancer compared to luminal breast cancer (*P* < 0.01). Kaplan–Meier survival analyses for recurrence-free survival (RFS) and particularly distant metastasis-free survival (DMFS) [17] demonstrated that the expression of ANXA1, FPR1 and FPR2 was negatively associated with the survival of lymph node positive patients with BRCA (Fig. 1b), indicating that indeed the high expression of ANXA1, FPR1 and FPR2 in metastatic patients may be linked with a significantly worse prognosis.

## Discussion

One of the major impediments to cure advanced breast cancer is the development of metastasis to the brain [16, 27]. Successful colonization of the brain is the ultimate outcome of an evolutionary process in which reciprocal interactions between metastatic cancer cells and the brain’s microenvironment, yield alterations that allow cancer cells to transcend their intrinsic characteristic [28, 29]. Yet, the biological processes that transform this hostility to permissivity for metastatic cancer cells to develop metastasis in the brain, remain an enigma. In addressing this challenge, our work links ANXA1-FPR1/2 and STAT3, as functional partners of a paracrine/autocrine loop, between metastatic mammary cancer cells and microglial cells in supporting metastatic colonization of mammary cancer in the brain.

Our study shows that the alteration of microglia from a cancer-fighting to a cancer-promoting phenotype, in the brain tumor microenvironment is an acquired trait, with tumor-associated microglia exhibiting an alternatively activated phenotype that produce anti-inflammatory factors such as TGF-β, IL-10 and ARG1 [30, 31]. This is similar to the M2 or alternative pro-tumor macrophage phenotype. The deterministic attribute of the metastatic cancer cell secretome, further explored in this study, revealed an extensive influence of soluble factors secreted by mammary cancer cells on microglia.

Experiments using the MMTV-Wnt transgenic mouse model for spontaneous breast cancer in this study provide evidence to further add another layer of complexity in the crosstalk between cancer cells and microglial cells in the brain. The findings from this model proposed that temporal evolution in changing a future metastatic brain tissue can occur earlier during primary tumorigenesis, than was previously thought [32]. The results also highlight that the microglia in the brain could be pre-conditioned as a result of the combined systemic effects of tumor-secreted factors produced by either primary or secondary mammary tumors in the periphery.

The majority of evidence from decades of tumor-associated macrophages (TAM) research has placed TAMs along a simple linear M1–M2 phenotypic continuum [33]. However, exposure of microglia to the tumor secretome deviated the dichotomous M1/M2 macrophage polarization scheme. Simultaneous up-regulation of gene expression for both pro-inflammatory and anti-inflammatory markers with tumor CM indicated that polarization induced by secretome of cancer cells is not absolute and may be relatively defined. The increased migratory phenotype, as well as, enhanced proliferation and activation of microglia induced metastatic breast cancer cells, suggests that chemokines/cytokines are among the soluble factors that constitute the secretome of metastatic mammary cancer cells. The opposing response of BV-2 microglia toward the secretome from 4T07 and 4T1 cells suggests that even within subtypes of breast cancer, cells with differential metastatic propensities can differ in their secretomic profile. In this case, reactive microglia in their pro-inflammatory capacity play a non-redundant role during chronic inflammation associated with early stages of tumorigenesis (non-metastatic). In the later stages of tumorigenesis (metastatic), microglia may promote disease progression in their pro-resolving capacity [34].

ANXA1 has been documented to be differentially expressed during the progression of breast cancer toward a more malignant state [12, 13]. In spite of its inherent roles in stimulating chemotaxis in immune cells and its pro-resolving properties, the implication of ANXA1 in secondary tumor microenvironment has not been rigorously investigated. Annexins, including ANXA1 are expressed on apoptotic cells and can bind to receptors such as dectin-1 on dendritic cells and microglia to regulate cytokine release and inflammation [35]. As anticipated, ANXA1 bestows upon 4T1 cancer cells, the ability for microglial recruitment. Intriguingly, genetic ablation of ANXA1 in 4T1 metastatic mammary cancer cells only modestly affect the pro- and anti-inflammatory gene expression profiles of BV-2 microglia stimulated by 4T1 CM.

Implicit in the disconnection between the two observations (treatment of BV-2 microglial cells with ΔANXA1 4T1 CM versus 4T1 CM with FPRs antagonists) is the interpretation that FPR1/2 was still activated despite genomic deletion of ANXA1 in 4T1 metastatic mammary cancer cells. Activation of FPR1/2 could still be achieved via two avenues, either by binding of the receptors with other agonists present in 4T1 CM or the presence of ANXA1 in our system secreted by microglia [36]. Our study found that ANXA1 from microglia was not required in the microglial migration induced by 4T1 CM.

An attempt to illustrate the role of ANXA1-FPRs axis in the crosstalk of metastatic mammary cancer cells and microglial cells, revealed that STAT3 as the downstream mediator of this interaction, working antagonistically to the ERK-STAT1 axis induced by 4T1 CM in BV-2 microglia. This is in line with other studies showing that ANXA1 can to FPR2 and regulate IL6 through STAT3 activation [25]. Although the activation of the MAPK pathways has been described downstream of FPRs activation, the present study stands out as an exception, as inhibition of FPRs activation led to a magnified activation of the ERK1/2-STAT1 pathway. This ERK-STAT1 pathway in microglia has only been reported by one recent study to contribute to bone cancer pain by regulating MHC class II expression in spinal microglia [37]. Cross-regulation of STAT1 and STAT3 has been reported by a number of studies, suggesting that STAT1 and STAT3 can compete for the same docking site on JAKs, same binding elements on DNA, or form STAT1/STAT3 heterodimers to limit the activity of STAT1 and STAT3 homodimers [38, 39]. Nevertheless, STAT1 activation is obligatory for anti-tumorigenic interferon signaling [40]. The antagonistic effect of STAT1 and STAT3 pathways in our study, confirms the established notion of the mutually antagonistic pathways of STAT1-driven anti-tumorigenic response, and STAT3-mediated immuno-suppression [41].

## Conclusion

**Fig. 8:**
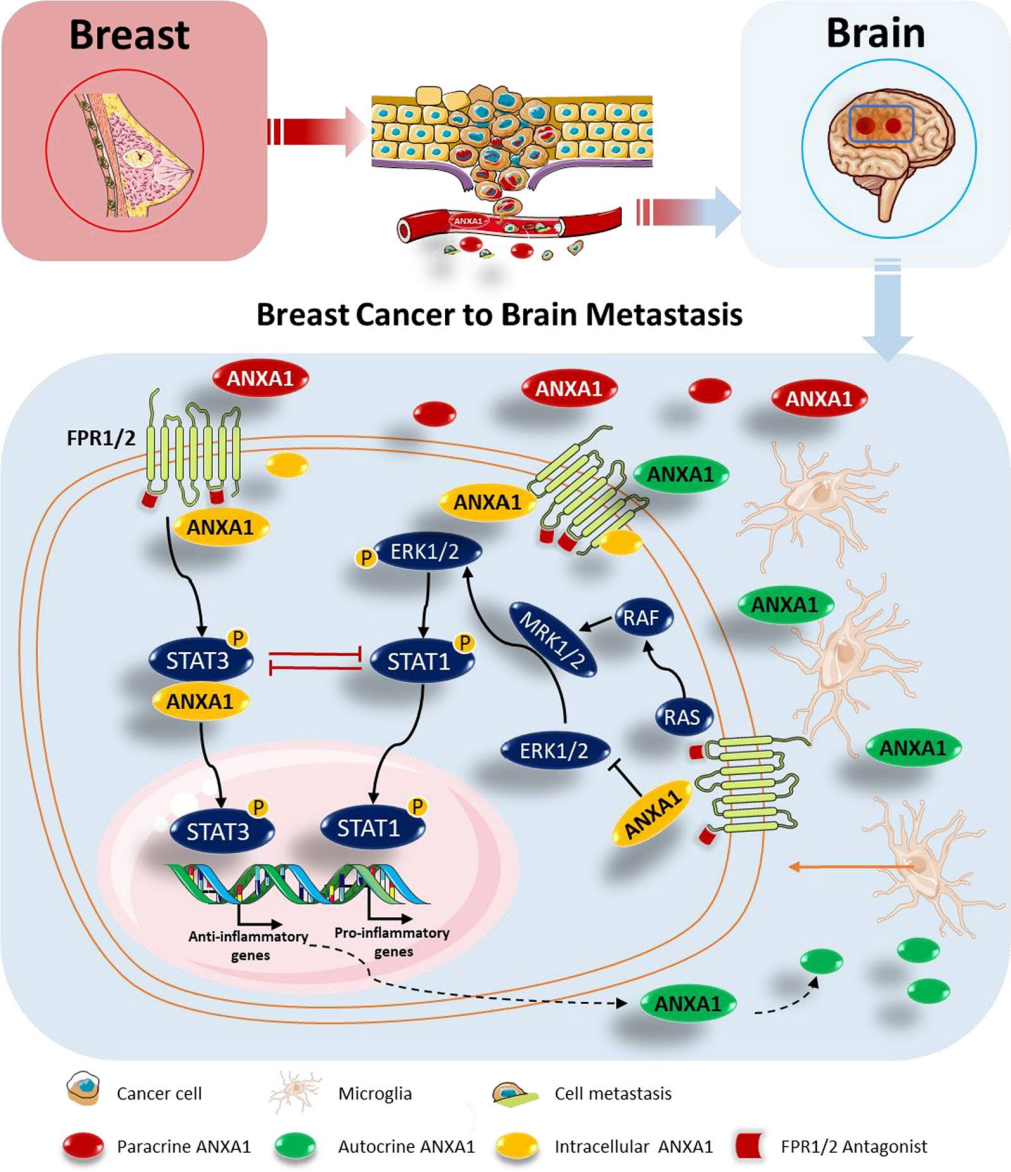
ANXA1-FPR-STAT3 and ERK1/2-STAT1 signaling in breast cancer to brain metastasis. ANXA1 is involved in an autocrine/paracrine signaling network between metastatic mammary cancer cells and microglial cells. Exogenous ANXA1 from tumors (red) or microglia (green) promotes microglial migration via regulation of the expression of several pro-inflammatory and anti-inflammatory genes, and this in turn, benefits cancer migration through its immune-modulatory effects on microglial cells via formyl-peptide receptors (FPRs) 1 and 2. Meanwhile, the endogenous ANXA1 expression (yellow) was triggered simultaneously in microglia, leading to enhanced activation of STAT3 induced by activated FPRs, and thus antagonizes the anti-tumorigenic ERK1/2-STAT1 pathway

As the progression of metastasis requires collusion between cancer cells and localized accumulations of myeloid cells, the findings in this study advocate that ANXA1 in the tumor microenvironment is partially responsible for recruitment of infiltrating microglia through activation of FPR1 and FPR2. As tumor evolves, the activation of ANXA1-FPR1/2-STAT3 axis in the microglia suppresses anti-tumor immunity through up-regulation of pro-tumorigenic factors, and down-regulation of anti-tumorigenic factors. The stable STAT3 feedforward loop is then maintained or enhanced by endogenous ANXA1 in microglial cells, contributing to synergistic effects on immune suppression and metastatic progression in the brain (Fig. 8).

A challenge for studies investigating the secretome of cancer cells is that the immuno-modulatory effects of the soluble factors present in the secretome could be context, time, concentration, and cell-type dependent. The use of conditioned media from ANXA1 knockout cell lines to elucidate the roles of ANXA1 on microglial cells could be confounded by the compensatory up-regulation or down-regulation of ANXA1-regulated genes. As ANXA1 is not the only agonist for FPRs, further validation experiments using ANXA1 protein is required to conclusively substantiate the roles of ANXA1. The functional role of ANXA1 in the tumor microenvironment should also be further validated by the use of neutralizing antibodies against ANXA1.

All in all, our work provides mechanistic insights into how microglia, in the brain tumor microenvironment, respond to metastatic breast cancer cells through activation of FPR1/2-STAT3 axis, driven by ANXA1 paracrine/autocrine loop. This cycle also sheds light on how ANXA1 is responsible for the infiltration of microglial cells into the tumor microenvironment and subsequently provides beneficial support to the survival of cancer cells facing the rigors of invading new microenvironments, leading to a clinical worse prognosis.

## Supplementary Information

**Additional file 1**.** Supplementary figure 1**. Activation of CD11b+CD45lo brain resident microglial cells in primary and secondary mammary tumors-bearing MMTV-Wnt1 mice.** Supplementary figure 2**. The expression pattern of ANXA1 in 4T1 and 4T07 cells.** Supplementary figure 3**. Characterisation of ANXA1 null 4T1 metastatic mammary cancer cells.** Supplementary figure 4**. ANXA1 in 4T1 metastatic mammary cancer cells secretome does not promote BV-2 microglial growth.** Supplementary figure 5**. FPRs antagonism attenuated gene expression of IL-6 and IL-10 Induced by 4T1 Metastatic Mammary Cancer Cells Conditioned Media.** Supplementary figure 6**. Metastatic mammary cancer cells secretome enhanced ANXA1 expression in microglia.** Supplementary figure 7**. Characterization of ANXA1 deletion in BV-2 microglial cells.** Supplementary figure 8**. Extracellular and intracellular ANXA1 elicited different effects on migratory and gene expression profiles of pro and anti-inflammatory markers in BV-2 microglial cells.** Supplementary figure 9**. Exogenous ANXA1 regulates STAT3 signalling through FPRs in microglia.** Supplementary figure 10**. Endogenous ANXA1 and MAPK activation in microglia.

## Data Availability

All data needed to evaluate the conclusions in the paper are present in the paper and/or the Additional file 1. Additional data related to this paper may be requested from the authors.
