# Supplementary material for: Breast cancer metastasis to brain results in recruitment and activation of microglia through annexin-A1/formyl peptide receptor signaling
Source: Breast Cancer Res. 2022 Apr 5;24:25. doi: 10.1186/s13058-022-01514-2 (PMC8985313; doi:10.1186/s13058-022-01514-2)
Supplement: Supplementary file 1 — Additional file 1. Supplementary figure 1. Activation of CD11b+CD45lo brain resident microglial cells in primary and secondary mammary tumors-bearing MMTV-Wnt1 mice. Supplementary figure 2. The expression pattern of ANXA1 in 4T1 and 4T07 cells. Supplementary figure 3. Characterisation of ANXA1 null 4T1 metastatic mammary cancer cells. Supplementary figure 4. ANXA1 in 4T1 metastatic mammary cancer cells secretome does not promote BV-2 microglial growth. Supplementary figure 5. FPRs antagonism attenuated gene expression of IL-6 and IL-10 Induced by 4T1 Metastatic Mammary Cancer Cells Conditioned Media. Supplementary figure 6. Metastatic mammary cancer cells secretome enhanced ANXA1 expression in microglia. Supplementary figure 7. Characterization of ANXA1 deletion in BV-2 microglial cells. Supplementary figure 8. Extracellular and intracellular ANXA1 elicited different effects on migratory and gene expression profiles of pro and anti-inflammatory markers in BV-2 microglial cells. Supplementary figure 9. Exogenous ANXA1 regulates STAT3 signalling through FPRs in microglia. Supplementary figure 10. Endogenous ANXA1 and MAPK activation in microglia. [file 13058_2022_1514_MOESM1_ESM.docx]

Additional file 1: for

**Breast cancer metastasis to brain results in recruitment and activation of microglia through Annexin-A1/formyl peptide receptor signaling**

Sok Lin Foo, Karishma Sachaphibulkij, Corinne L.Y. Lee, Gracemary L.R. Yap, Jianzhou Cui, Thiruma Arumugam, Lina H.K. Lim^*^

*Corresponding author. Lina H K Lim, [linalim@nus.edu.sg](mailto:linalim@nus.edu.sg)

**This PDF file includes:**

**Supplementary Text**

**Figs. S1 to S10**

**Table S1**

**Additional file 1: Text**

**Materials and Methods**

# Preparation of Tumour Cell Conditioned Media

4T1 and 4T07 murine mammary cancer cells were cultured in 10 mL of DMEM/F12 culture media supplemented with 10% heat-inactivated FBS, 1mM L-glutamine,1mM sodium pyruvate, 100 U/mL penicillin, and 100 μg/mL streptomycin in 75 cm^2^ cell culture flasks (Cellstar, Greiner Bio-one, Gloucestershire, UK) in a humidified incubator with 5% CO^2^ at 37°C. The cells were allowed to reach 80-90% confluency before the media was removed and replaced with plain fresh media. Cell culture supernatant was harvested from adherent cancer cells after 24 hours. The supernatant was centrifuged, filtered through a syringe filter membrane (0.2 µm) (Sartorius), and stored at −80°C until use as conditioned media (CM).

# Haematoxylin and Eosin Staining

Slide was first baked at 60 ^o^C for 15-20 min before deparaffinising step using xylene. Slide was rehydrated using 100% ethanol, followed by 95% ethanol and 70% ethanol accordingly. Tap water was used to rinse the slide before proceeding to hematoxylin staining. Dried slide was dipped into Mayer’s Hematoxylin solution for 1-5 min. It was next dipped into acid ethanol for destaining. Before dipping the slide few times in Bluing reagent, slide was rinsed with warm tap water. Slide was placed in reagent alcohol 95%, followed with dipping into 1% Eosin Y for 0.5-2 min with agitation. 95% and 100% ethanol was used to dehydrate eosin-stained slide. Slide was next cleared with three rounds of Xylene solution and was dried. After mounting coverslip on the slide, the coverslip was sealed with sealant and the slide was left dried overnight.

# *In vivo* Models of Breast Cancer Brain Metastasis

1. **MMTV-Wnt transgenic model of metastatic breast cancer with primary tumour resection.** MMTV-Wnt transgenic mice were bred and monitored for development of palpable primary tumours ≥1.5cm in diameter. Surgery was performed for primary tumour resection and the mice were allowed to recover. Upon development of relapse, mice were euthanized and the brains were harvested for brain cells isolation. Tumour volume was calculated as (length × width × width/2).
2. **4T1-Balb/c intracarotid injection brain metastasis model.** Wild type and ANXA1-/- Balb/c mice were randomized into two groups each (6 mice per group). Mice were anaesthetized with isoflurane and the right common carotid artery of the mice was carefully exposed and separated from vagal nerve. After tying one end loosely with a surgical ligature, one external carotid artery branch was tied tightly with another surgical ligature. By using a small syringe with a 32 G needle, 50 μL of stably transfected 4T1-luciferase cell suspension (10^4^ cells) was injected into the internal carotid artery. The loosely tied ligature on the common carotid artery was removed and the wound was sealed. Bioluminescence imaging was performed to monitor tumour growth. Mice were euthanized and the brains were harvested for brain cells isolation at the end of the study when significant weight loss or signs of distress was observed.

# *In vivo* Bioluminescence Imaging. The location and growth size of the tumours and tissue metastasis were monitored by a bioluminescence-imaging assay. Mice were injected with 150mg/kg body weight of 15mg/mL VivoGlo^TM^ Luciferin (Promega, USA) dissolved in Dulbecco's phosphate-buffered saline (DPBS) (GE Healthcare Life Sciences, USA) before imaging with Xenogen IVIS Spectrum Imaging System (Caliper Life Sciences, USA).

## Generation and characterization of ANXA1 deletion in 4T1 metastatic mammary cancer cells

To investigate the role of ANXA1 externalized by 4T1 metastatic mammary cancer cell on BV-2 microglial cells, CRISPR-Cas9 genome editing system was used to carry out genomic deletion of ANXA1 in 4T1 metastatic mammary cancer cells. ANXA1 deletion was assessed by Western blot analysis and qRT-PCR, with the expression levels of ANXA1 normalized against GAPDH levels. As shown by Western blots analysis, five ANXA1 null clones (clone 1, 3, 4, 7 and 8) were generated in 4T1 metastatic mammary cancer cells **(Fig. S3A).** Clone 1 was selected for subsequent experiments. The cells were also assessed for morphological change following confirmation of ANXA1 deletion from their genome. ΔANXA1 4T1 metastatic mammary cancer cells demonstrated a more epithelium-like phenotype, with the cells attaching to each other to form colonies while proliferating (**Fig. S3B**). As expected, there was negligible gene expression of ANXA1 in ΔANXA1 4T1 metastatic mammary cancer cells when compared to WT 4T1 cancer cells (*p=0.0010*) **(Fig. S3C).** Proliferation assay also showed that ANXA1 plays a role in cell proliferation of cancer cells as ΔANXA1 4T1 cells grew significantly slower than their WT counterparts, with significant difference observed at 72-hr (0.65-fold, *p<0.0001*) and 96-hr (0.28-fold, *p=0.0085*) (**Fig. S3D**).

## Generation and Characterization of ANXA1 deletion in BV-2 Microglial Cells

To dissect the contribution of ANXA1 from microglial cells to the observed effects, ΔANXA1 BV-2 microglial cell line was generated using CRISPR/Cas9 genome-editing system with gRNA for ANXA1. ANXA1 deletion was assessed by Western blot analysis, with the expression levels normalized against GAPDH levels. Two ΔANXA1 BV-2 clones were generated (clone 1 and 2) and clone two was selected for subsequent experiments **(Fig. S7A).** Morphologically, ΔANXA1 BV-2 displayed more swollen cell bodies compared to WT **(Fig. S7B).** As confirmed by quantitative RT-PCR analysis, gene expression of ANXA1 was negligible in clone two of the ΔANXA1 BV-2 microglial cells compared to WT BV-2 microglial cells (*p=0.0257*) **(Fig. S7C).** However, the proliferation rate of ΔANXA1 BV-2 was not different compared to their WT counterparts **(Fig. S7D).**


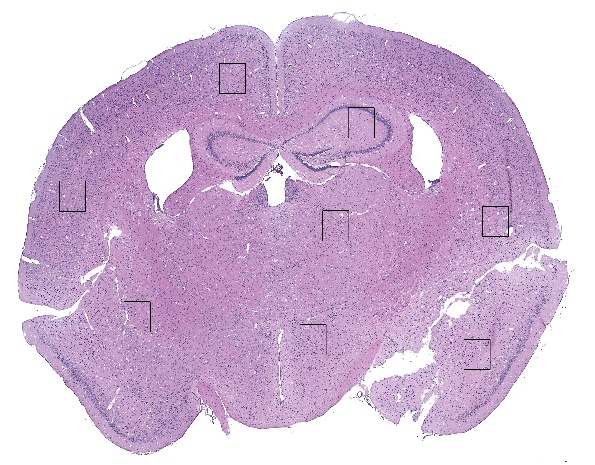


a

b

c

d

e

f

g

h


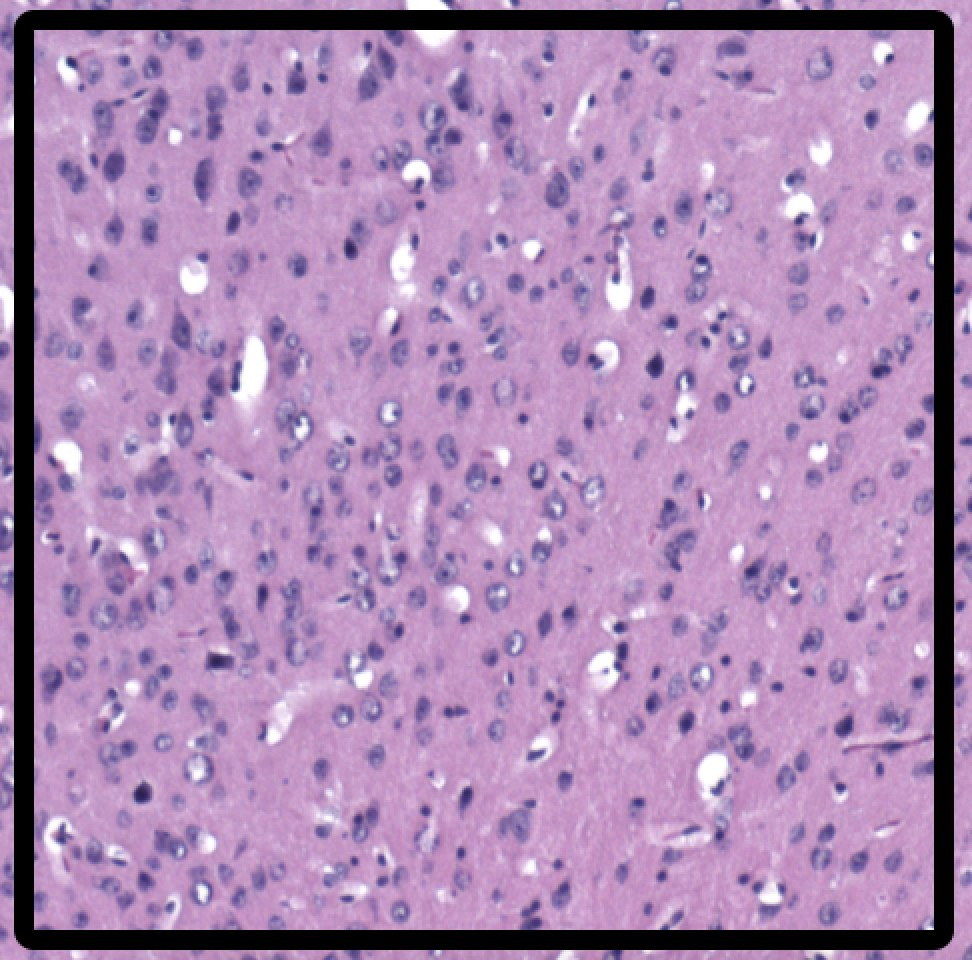


a


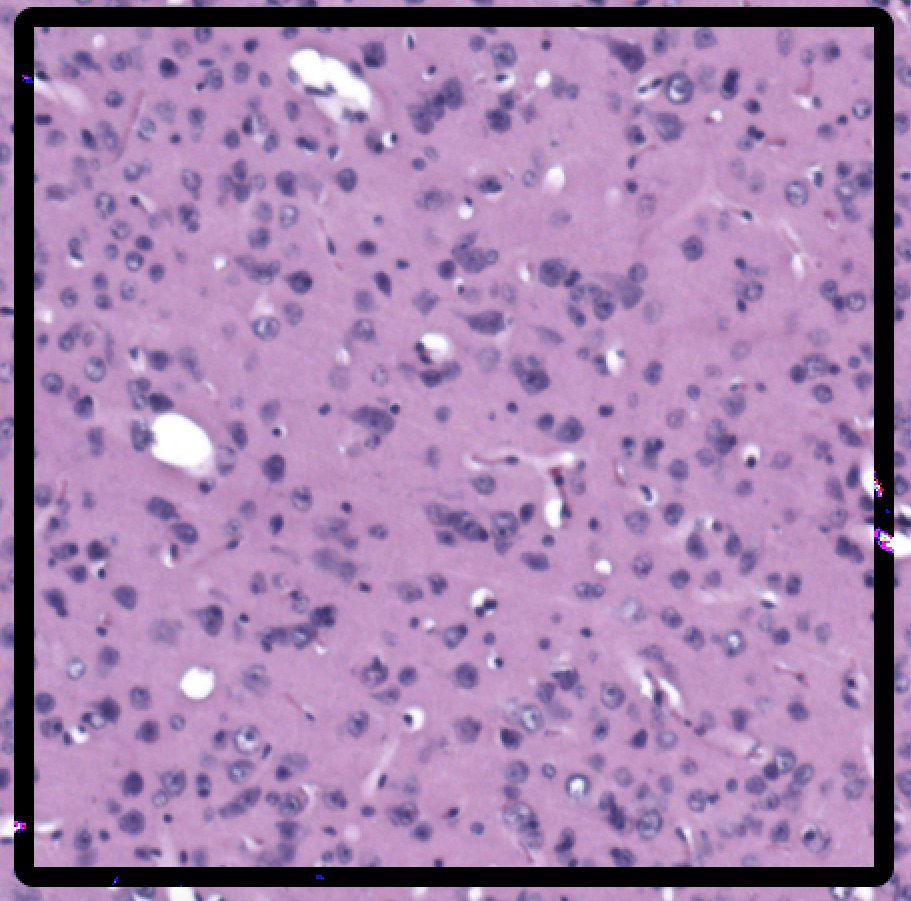


b


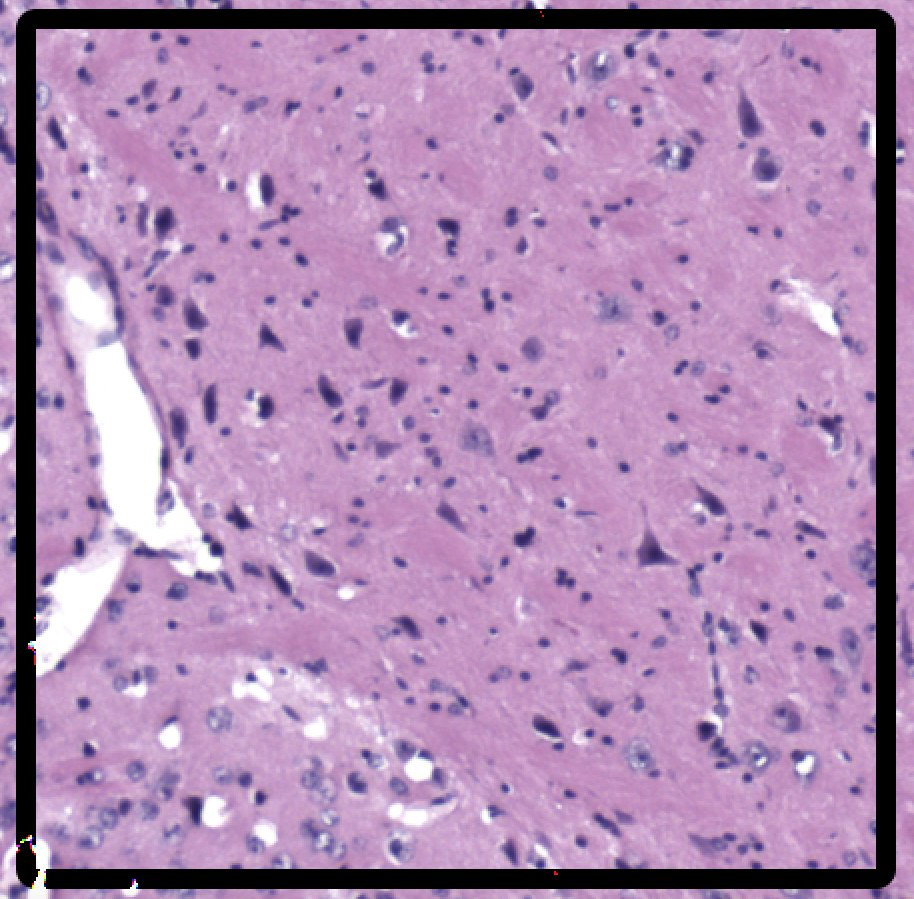


c


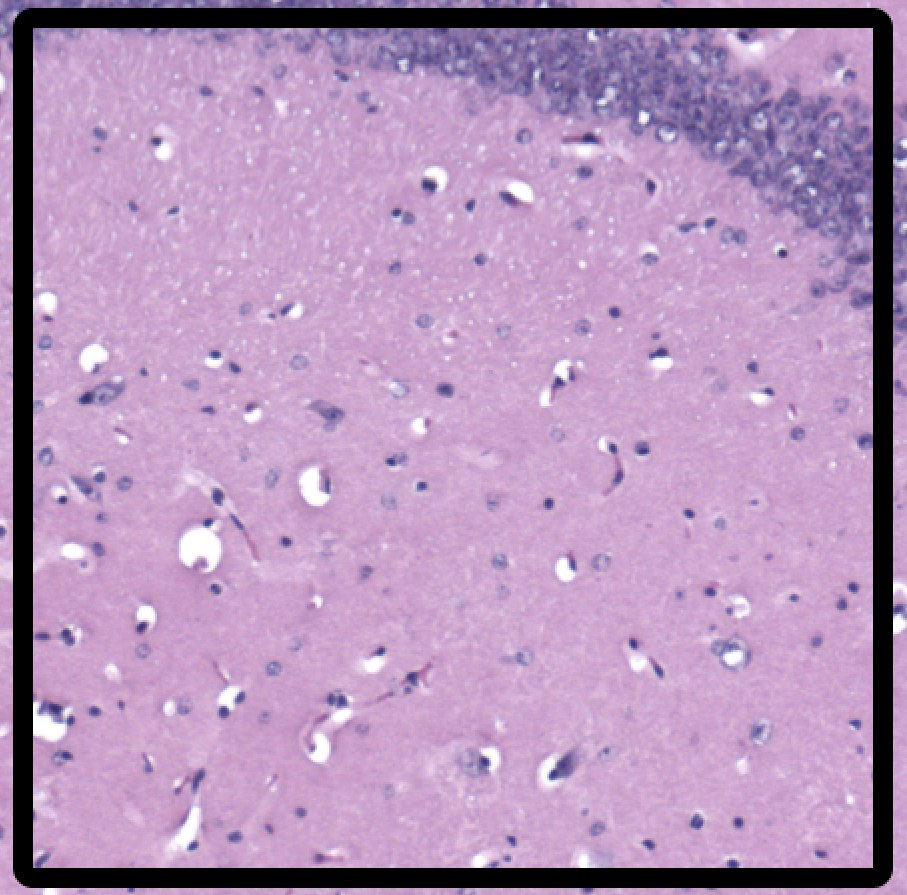


d


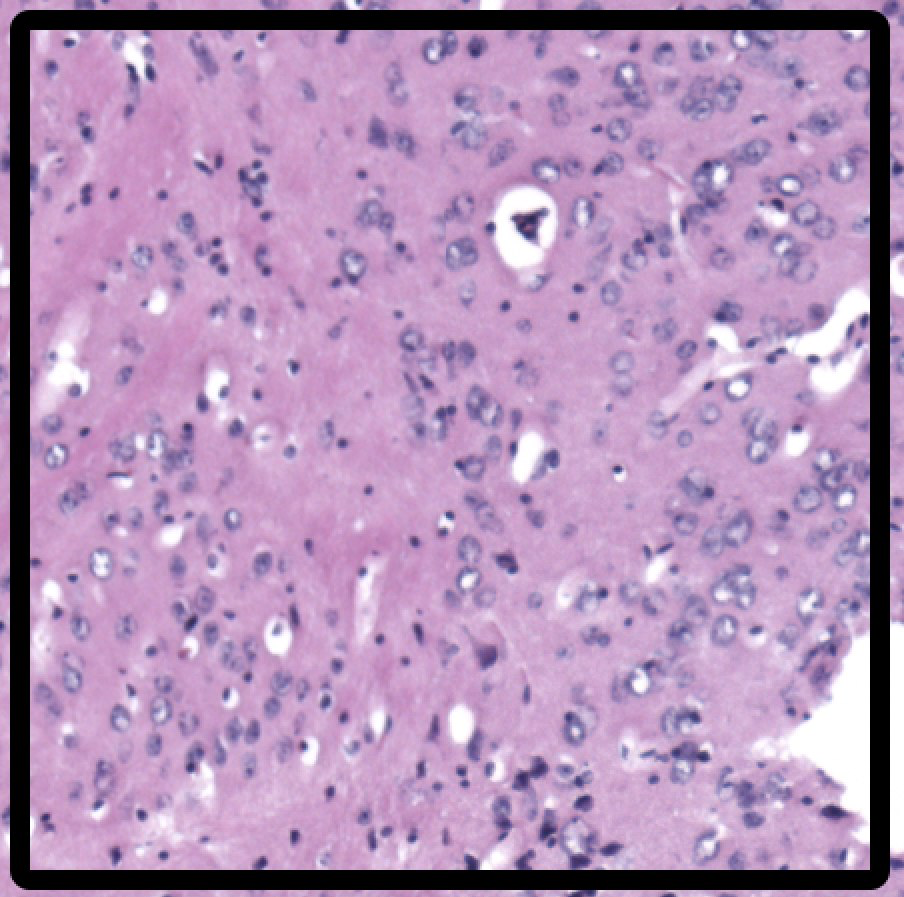


e


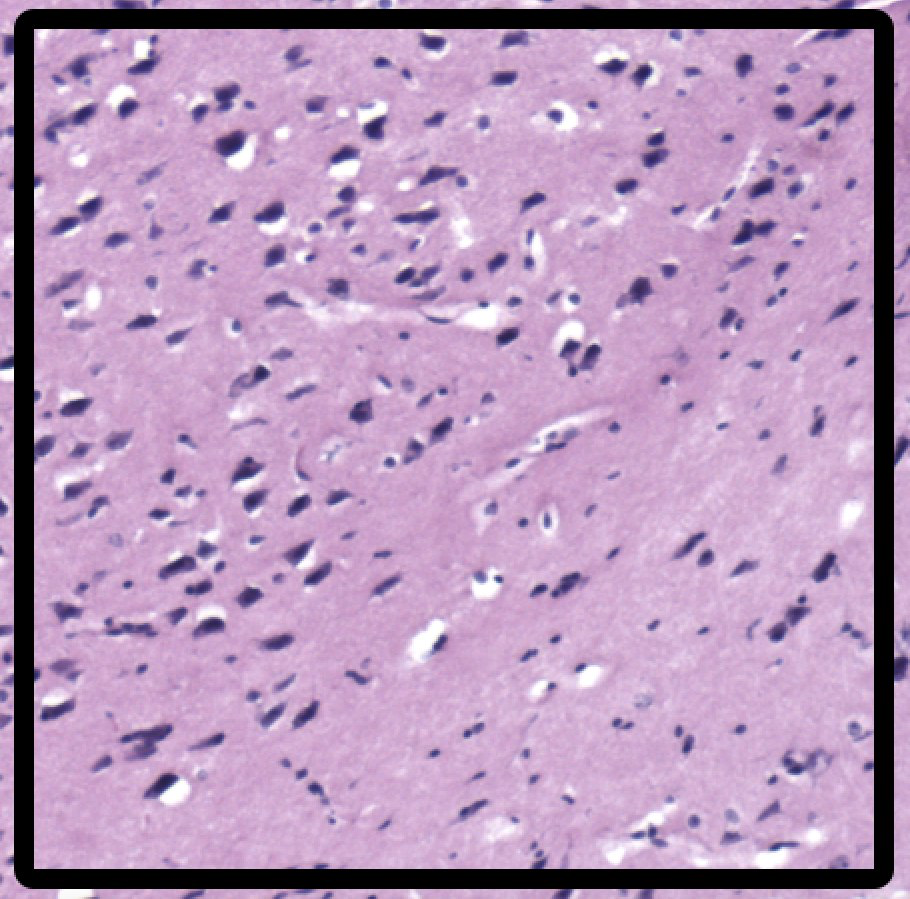


f


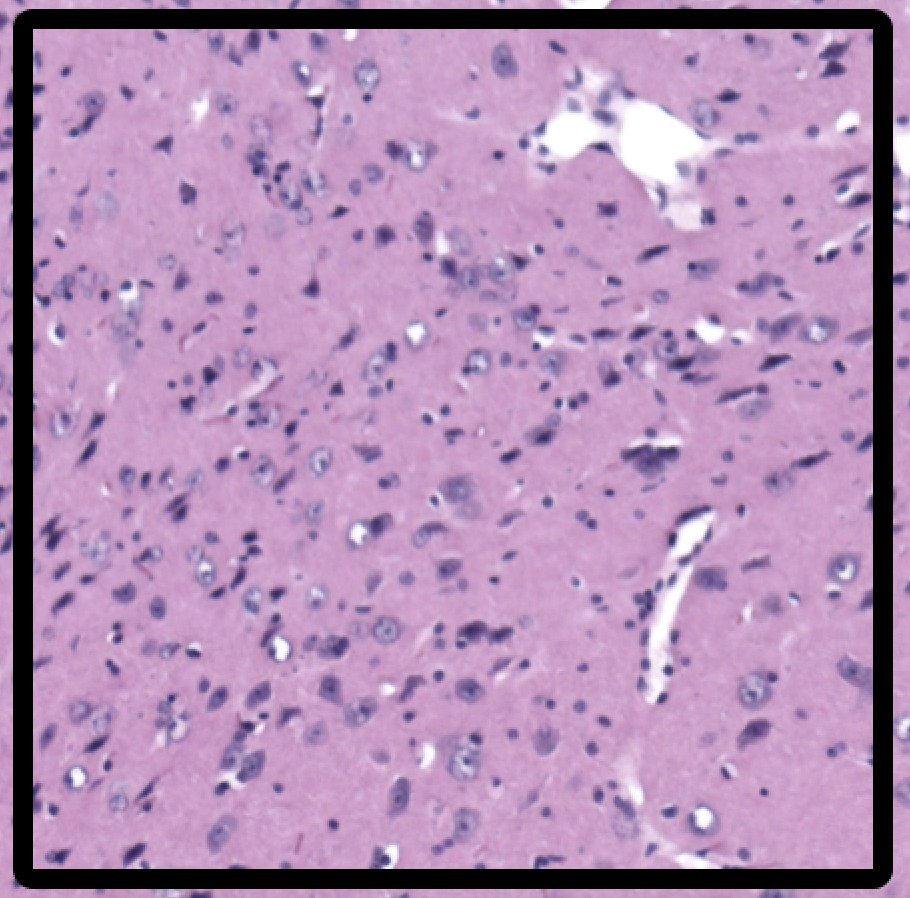


g


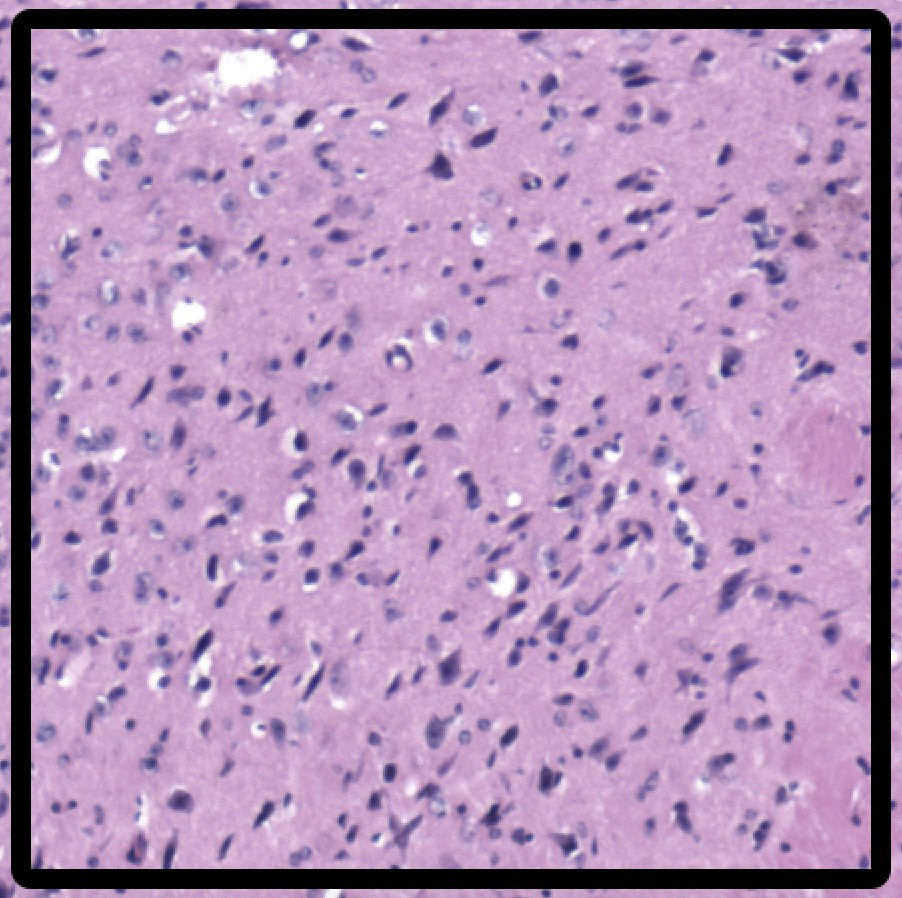


h

**A**


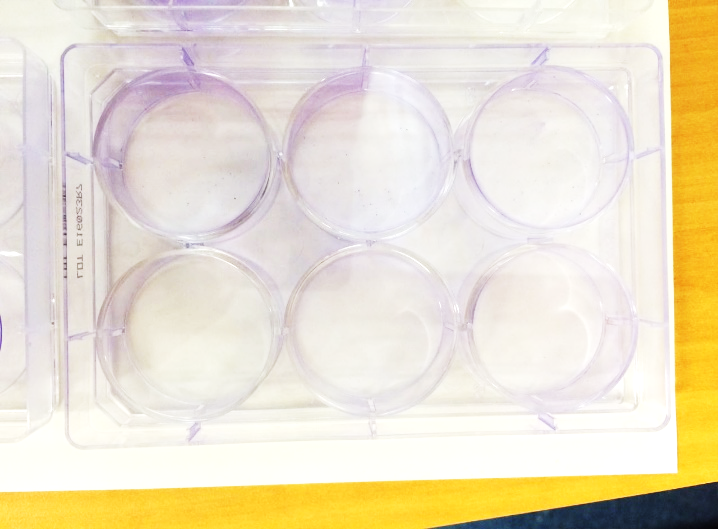

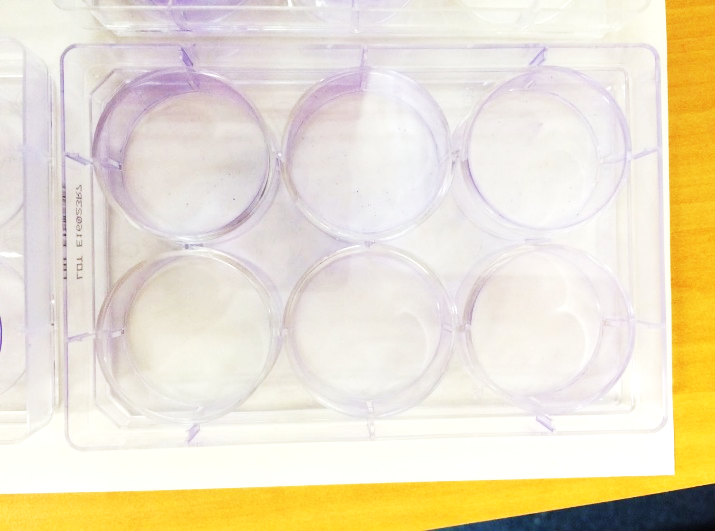

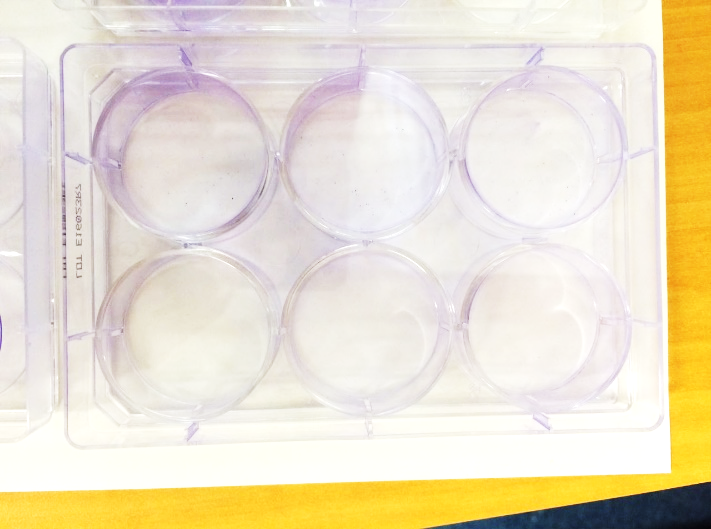


**B**

**Additional file 1: Fig. S1. Activation of CD11b^+^CD45^lo^ brain resident microglial cells in primary and secondary mammary tumors-bearing MMTV-Wnt1 mice. (A)** Representative images of tissue staining by haematoxylin and eosin of formalin-fixed, paraffin-embedded brain section from MMTV-Wnt1 mouse bearing secondary tumor in the periphery between day 14 to day 21 after primary tumor resection (left) (n=5 mice/group). Right: Magnified images (3x magnification) of brain section labelled a-h from image on the left. Scale bar: 1 mm. (**B)** Clonogenic assay of MMTV-Wnt1 brains bearing secondary tumors in the periphery between day 14 to day 21 after primary tumor resection (n=5 mice/group).


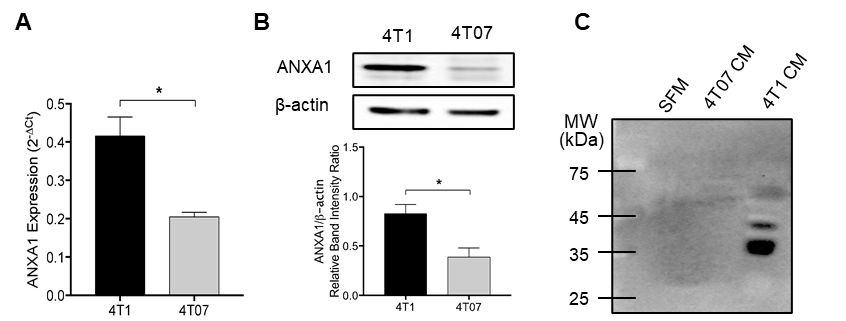


**Additional file 1: Fig. S2. The expression pattern of ANXA1 in 4T1 and 4T07 cells. (A)** Gene expression of ANXA1 in 4T1 metastatic mammary cancer cells compared to 4T07 non-metastatic mammary cancer cells determined by qRT-PCR analysis. **(B)** Western-blot analysis showing ANXA1 expression in 4T1 metastatic mammary cancer cells or 4T07 non-metastatic mammary cancer cells. Loading control: β-actin. Graph: densitometric quantification of the relative levels of ANXA1 protein expression in the cells. **(C)** Western-blot analysis of ANXA1 protein in either SFM, 4T07 CM or 4T1 CM. Data representative of three independent experiments.

**Additional file 1: Fig. S3. Characterisation of ANXA1 null 4T1 metastatic mammary cancer cells. (A)** Representative immunoblot showing protein expression of ANXA1 in WT and ΔANXA1 4T1 metastatic mammary cancer cells. β-actin was detected for loading control. **(B)** Representative bright field images of morphology of WT and ΔANXA1 4T1 metastatic mammary cancer cells (100x magnification). **(C)** Gene expression of ANXA1 determined by quantitative RT-PCR, normalized to the housekeeping gene GAPDH in WT and ΔANXA1 4T1 metastatic mammary cancer cells. **(D)** Time-dependent (0-96 hours) proliferation of WT and ΔANXA1 4T1 metastatic mammary cancer cells. Data represents mean ± standard error of the mean (SEM) of an n of three. **C**, P-values from unpaired two-tailed t-test. **D**, P-values from two-way ANOVA, Tukey’s multiple comparison post-hoc test.


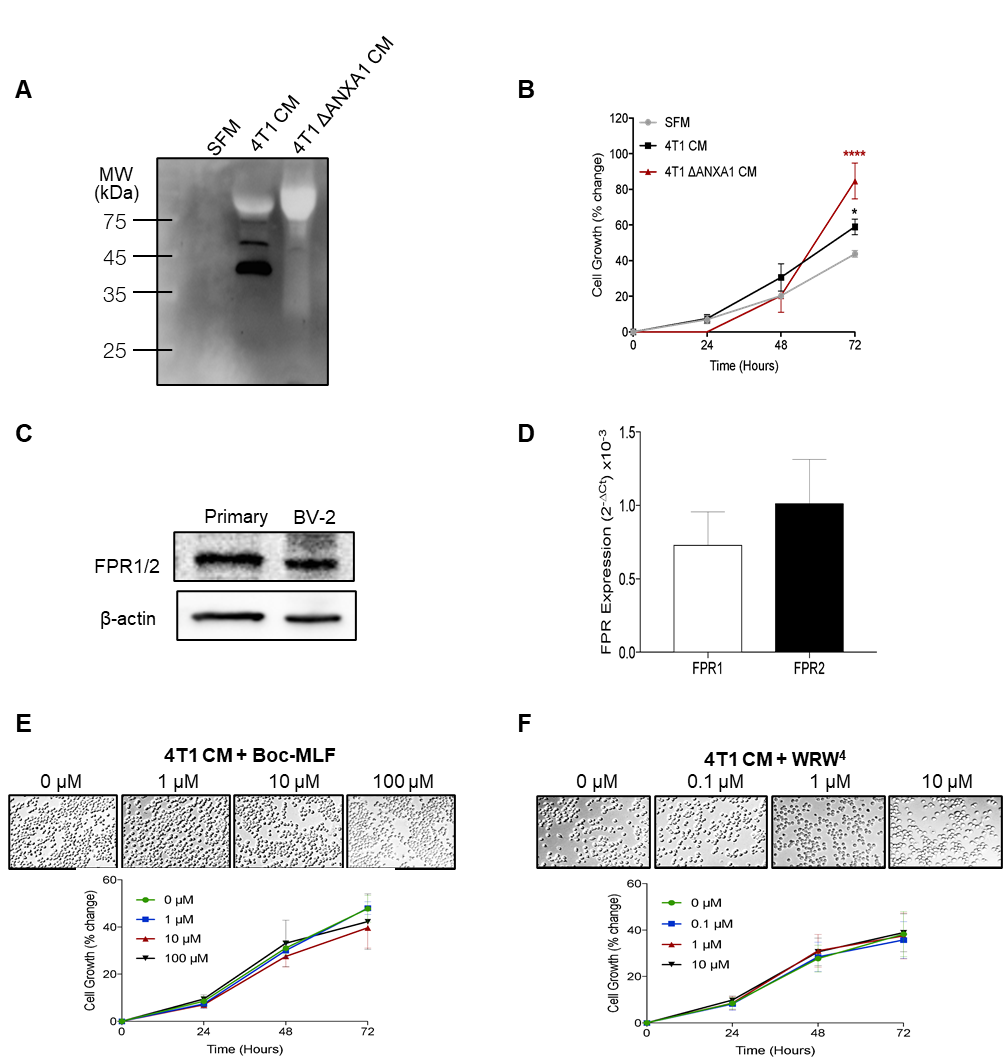


**Additional file 1: Fig. S4. ANXA1 in 4T1 metastatic mammary cancer cells secretome does not promote BV-2 microglial growth.** (**A**) Western-blot analysis of ANXA1 protein in either SFM, 4T1 CM or in ΔANXA1 4T1 CM. Data representative of three independent experiments. (**B**) Growth curves of BV-2 microglial cells treated with either SFM, 4T1 CM or 4T1 ΔANXA1 CM for 72 h. (**C**) Western-blot analysis showing protein expression of FPRs in murine adult primary and BV-2 microglial cells. Loading control: β-actin. (**D)** Gene expression of FPR1 and FPR2 in BV2 microglial cells determined by qRT-PCR analysis. Critical threshold (CT) values were subtracted from that of GAPDH, averaged and converted from log-linear to linear term. (**E)** Top: representative brightfield images of BV-2 microglial cells treated with 4T1 CM supplemented with either 0, 1, 10, or 100 μM of Boc-MLF FPR1 antagonist at 72 hr. Bottom: Growth curves of BV-2 microglial cells under the same treatments for 72 h. (**F)** Top: representative brightfield images of BV-2 microglial cells treated with 4T1 CM supplemented with either 0, 0.1, 1, or 10 μM of WRW4 FPR2 antagonist at 72 hr. Bottom: Growth curves of BV-2 microglial cells under the same treatments for 72 hr.


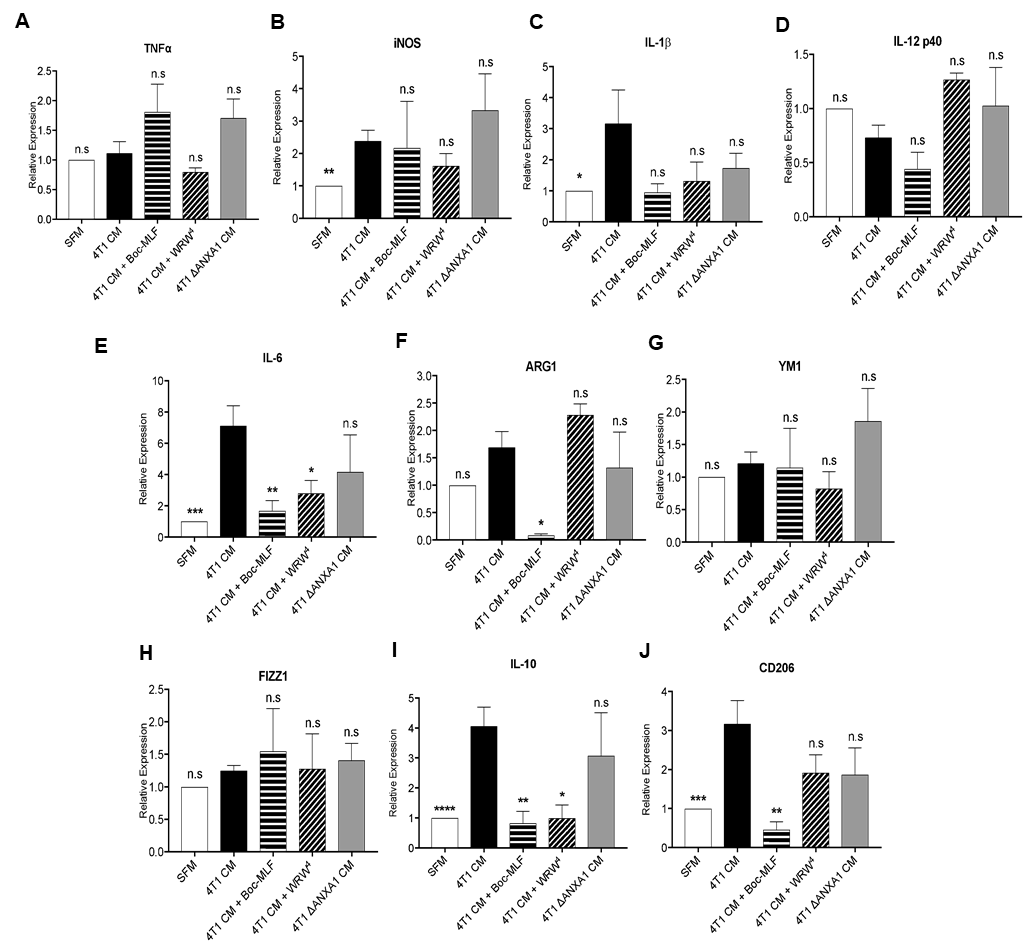


**Additional file 1: Fig. S5. FPRs antagonism attenuated gene expression of IL-6 and IL-10 Induced by 4T1 Metastatic Mammary Cancer Cells Conditioned Media. (A-E)** Gene expression of pro-inflammatory markers, TNFα (A), iNOS (B), IL-1β (C), IL-6 (D), and IL-12 p40 (E), determined by qRT-PCR analysis from BV-2 microglia treated with either SFM, 4T1 CM, 4T1 CM + Boc-MLF, 4T1CM + WRW4, or 4T1 ΔANXA1 CM. **(F-J)** Gene expression of anti-inflammatory markers, ARG1 (F), YM1 (G), FIZZ1 (H), IL-10 (I), and CD206 (J), determined by qRT-PCR analysis from BV-2 microglia treated with either SFM, 4T1 CM, 4T1 CM + Boc-MLF, 4T1CM + WRW4, or 4T1 ΔANXA1 CM. Data represent mean ± SEM; n = 3 independent experiments. *P < 0.05, **P < 0.01, ***P < 0.001, ****P < 0.0001.


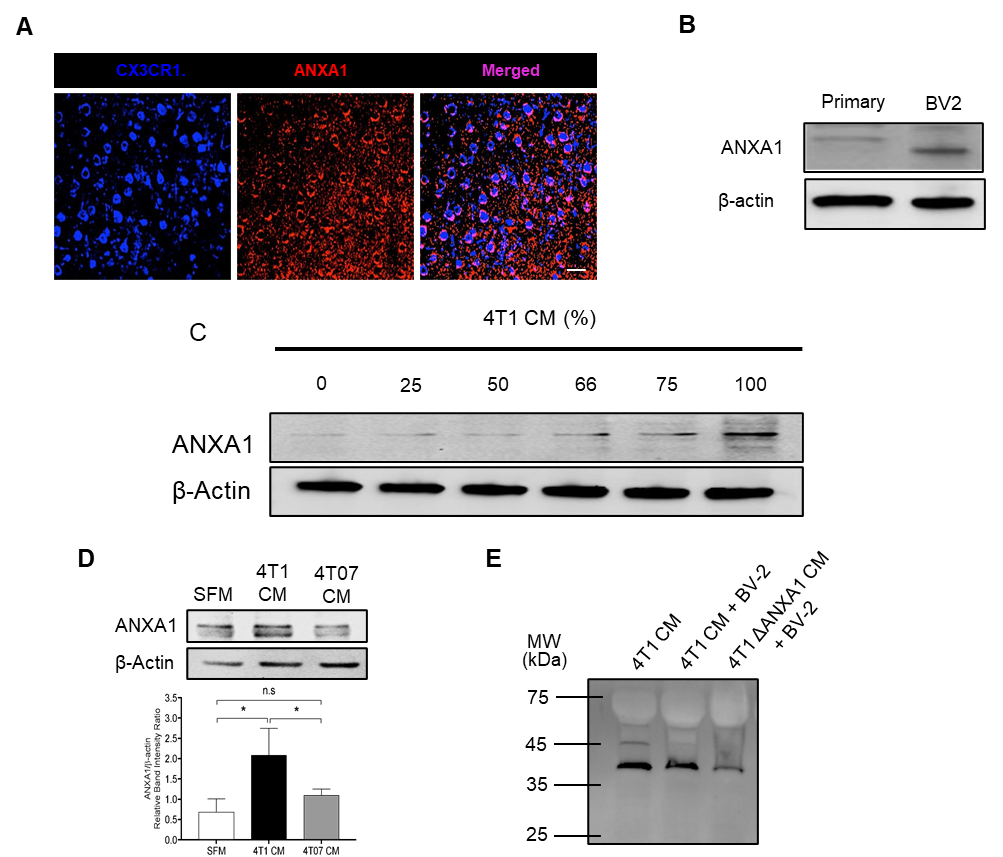


**Additional file 1: Fig. S6. Metastatic mammary cancer cells secretome enhanced ANXA1 expression in microglia. (A)** Representative images of immunofluorescence labelling of ANXA1 (red) and microglial marker CX3CR1 (blue) in brain section of 6-month-old WT C57/BL6 mouse. Merged image is shown on the right. (**B)** Western blot analysis showing ANXA1 expression in primary adult microglia and BV-2 microglial cell line. Loading control: β-actin. (**C)** Western blot analysis showing ANXA1 expression in BV-2 microglial cells treated with either 0, 25, 50, 66, 75 or 100% of 4T1 CM in SFM mixture for 24 h. Loading control: β-actin. **(D)** Western-blot analysis showing ANXA1 expression in BV-2 microglia treated with either SFM, 4T1 CM or 4T07 CM. Loading control: β-actin. Graph: densitometric quantification of the relative levels of ANXA1 protein expression in the cells. **(E)** Western-blot analysis of ANXA1 protein in either 4T1 CM, supernatant of BV-2 microglial cells treated with 4T1 CM, or supernatant of BV-2 microglial cells treated with 4T1 ΔANXA1 CM. Data represent mean ± SEM; n = 3 independent experiments. Images are representative of three independent experiments. P-values from one-way ANOVA with Tukey´s multiple comparison test. *P < 0.05.


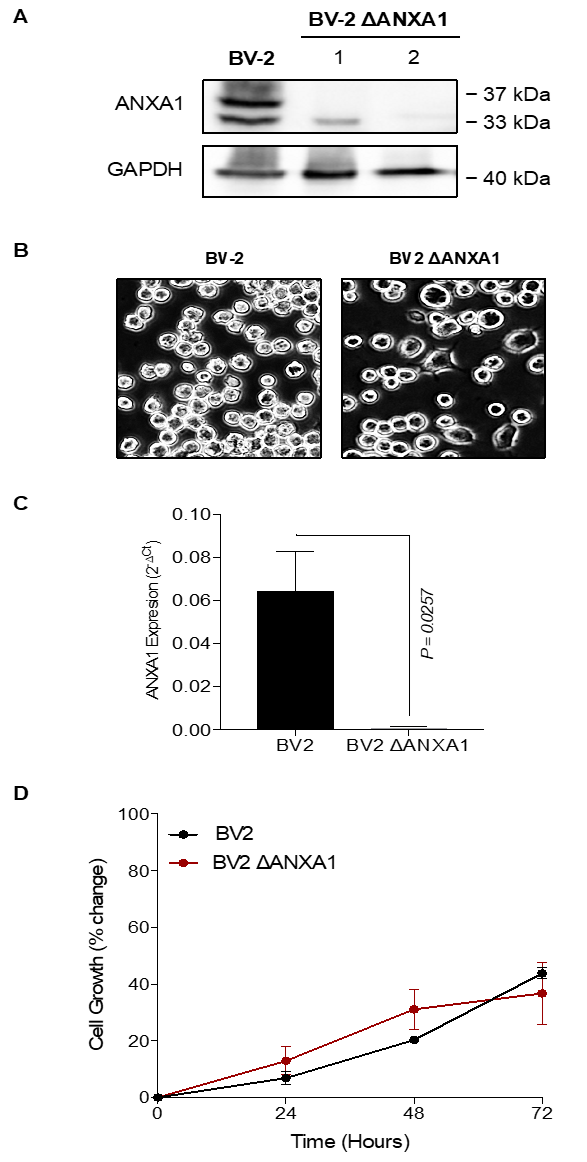


Additional file 1: Fig. S7. Characterization of ANXA1 deletion in BV-2 microglial cells.

**(A)** Representative immunoblot showing protein expression of ANXA1 in WT and ΔANXA1 BV-2 microglial cells. GAPDH was detected for loading control. **(B)** Representative bright field images of morphology of WT and clone 2 of ΔANXA1 BV-2 microglial cells (100x magnification).**(C)** Gene expression of ANXA1 determined by quantitative RT-PCR, normalized to the housekeeping gene GAPDH in WT and clone 2 of ΔANXA1 BV-2 microglial cells. **(D)** Time-dependent (0-72 hours) proliferation of WT and ΔANXA1 BV-2 microglial cells. Data represents mean ± standard error of the mean (SEM) of an n of three. **C**, P-values from unpaired two-tailed t-test. **D**, P-values from two-way ANOVA, Tukey’s multiple comparison post-hoc test.

**C**


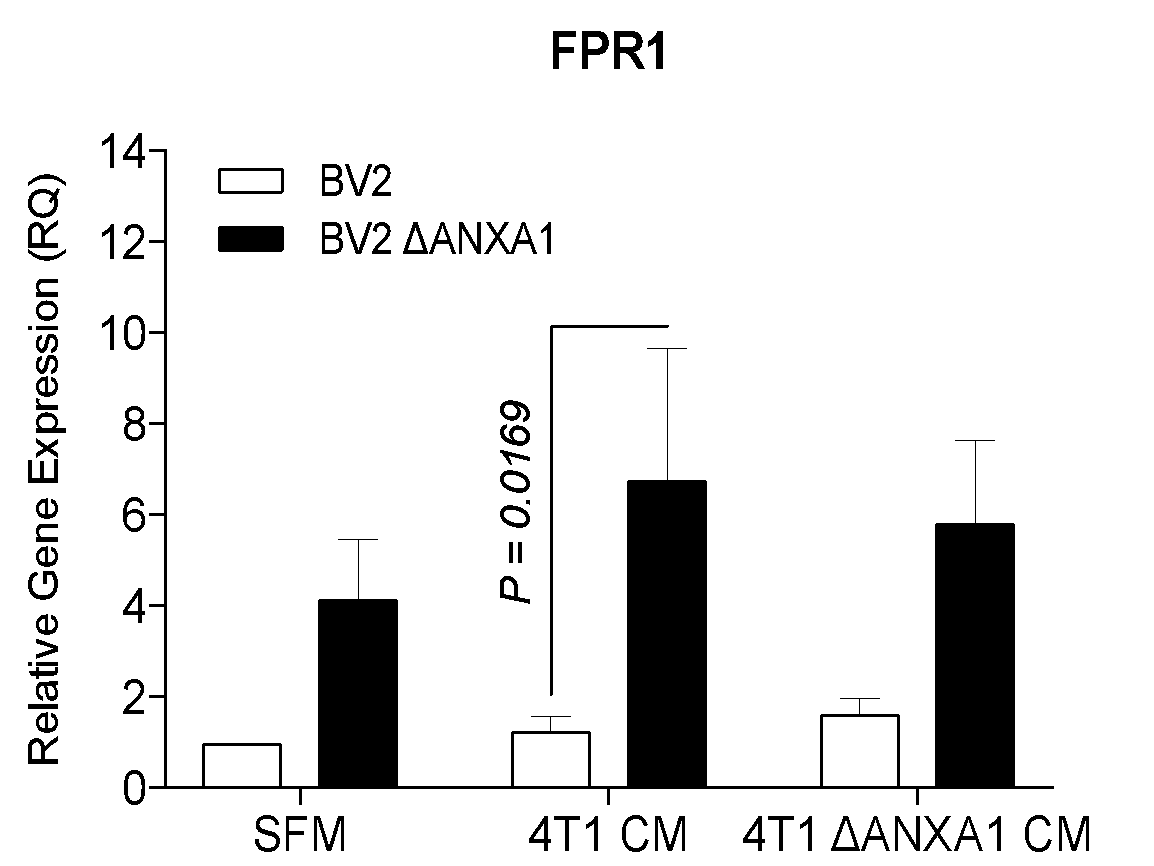


**D**


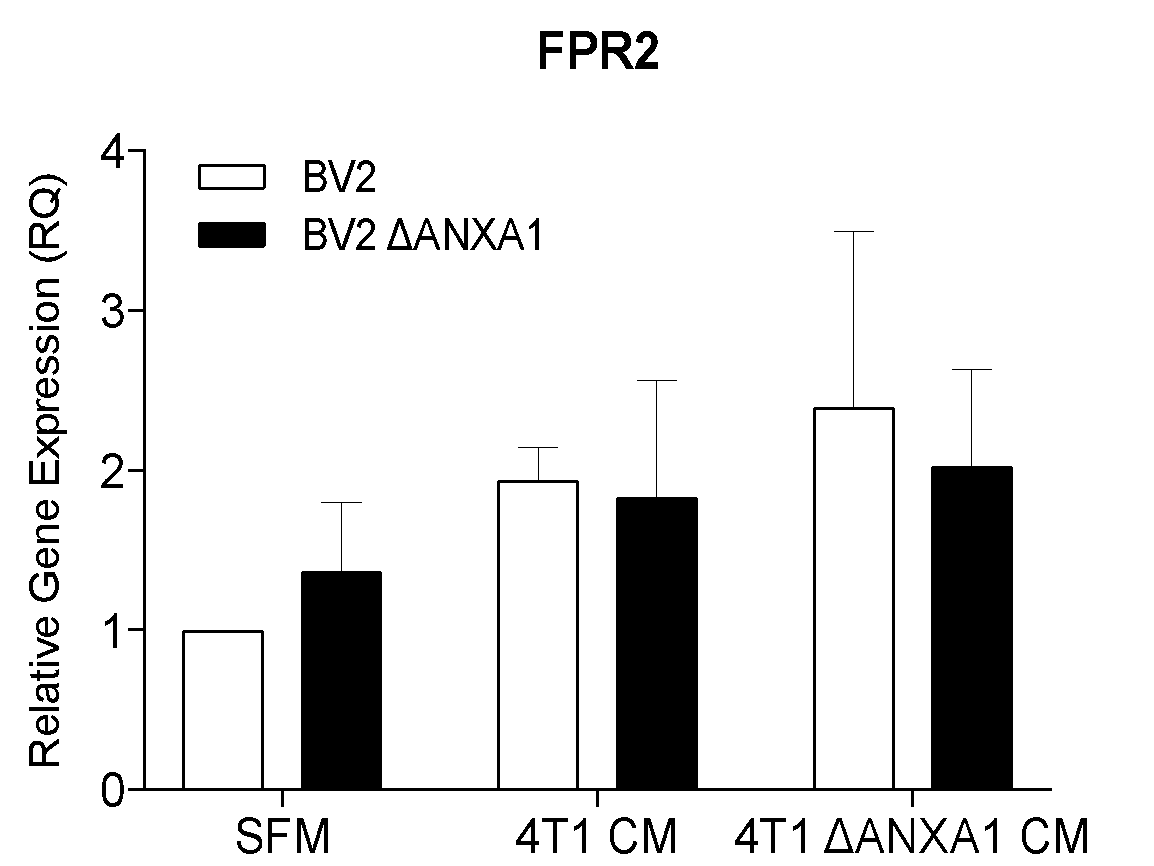


**A**


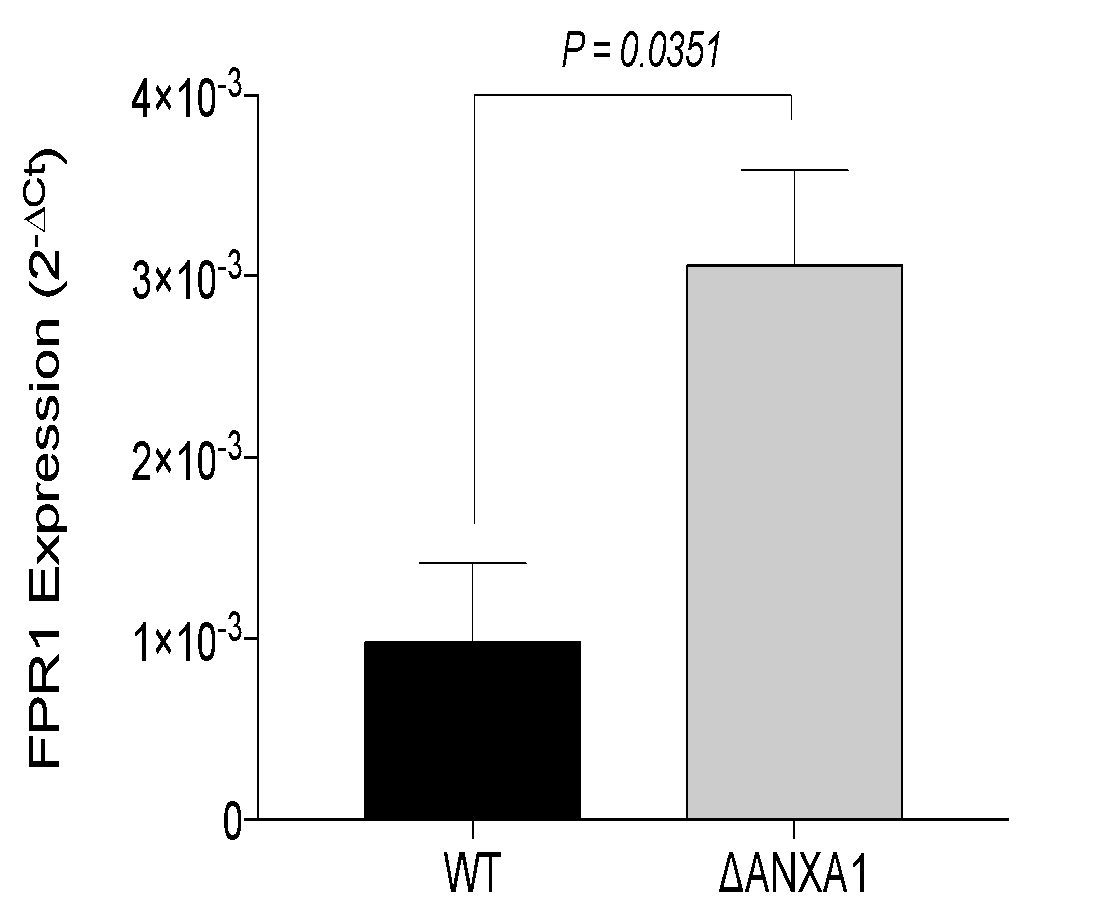


**B**


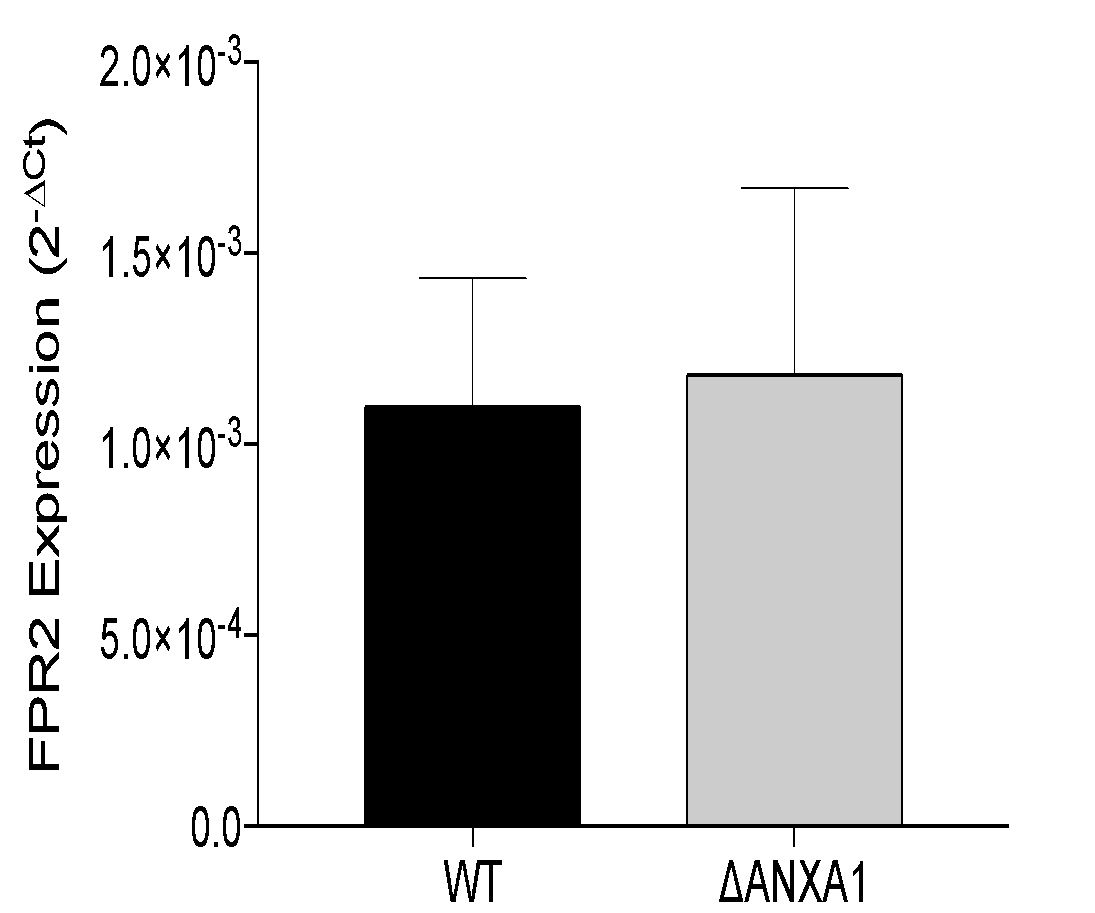

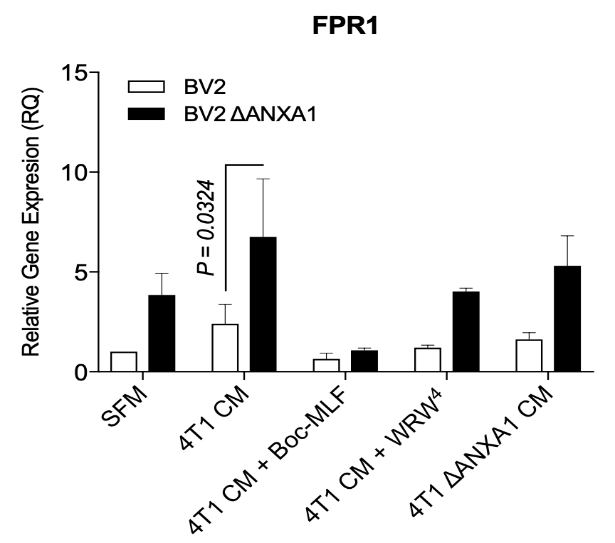


**E**

**Additional file 1: Fig. S8. Extracellular and intracellular ANXA1 elicited different effects on migratory and gene expression profiles of pro and anti-inflammatory markers in BV-2 microglial cells. (A, B).** Gene expression of FPR1 and FPR2 in WT and ΔANXA1 BV-2 microglial cells determined by qRT-PCR analysis. **(C, D)** Gene expression of FPR1and FPR2 determined by qRT-PCR analysis from BV-2 and BV-2 ΔANXA1 microglia treated with either SFM, 4T1 CM. **(E)** Gene expression of FPR1 determined by qRT-PCR analysis from BV-2 and BV-2 ΔANXA1 microglia treated with either SFM, 4T1 CM, 4T1 CM + Boc-MLF, 4T1CM + WRW4. Data represent mean ± SEM; n = 3 independent experiments. P-values from unpaired two-tailed *t*-test (A and B), two-way ANOVA, Sidak’s multiple comparison post-hoc test (C-E). *P < 0.05.


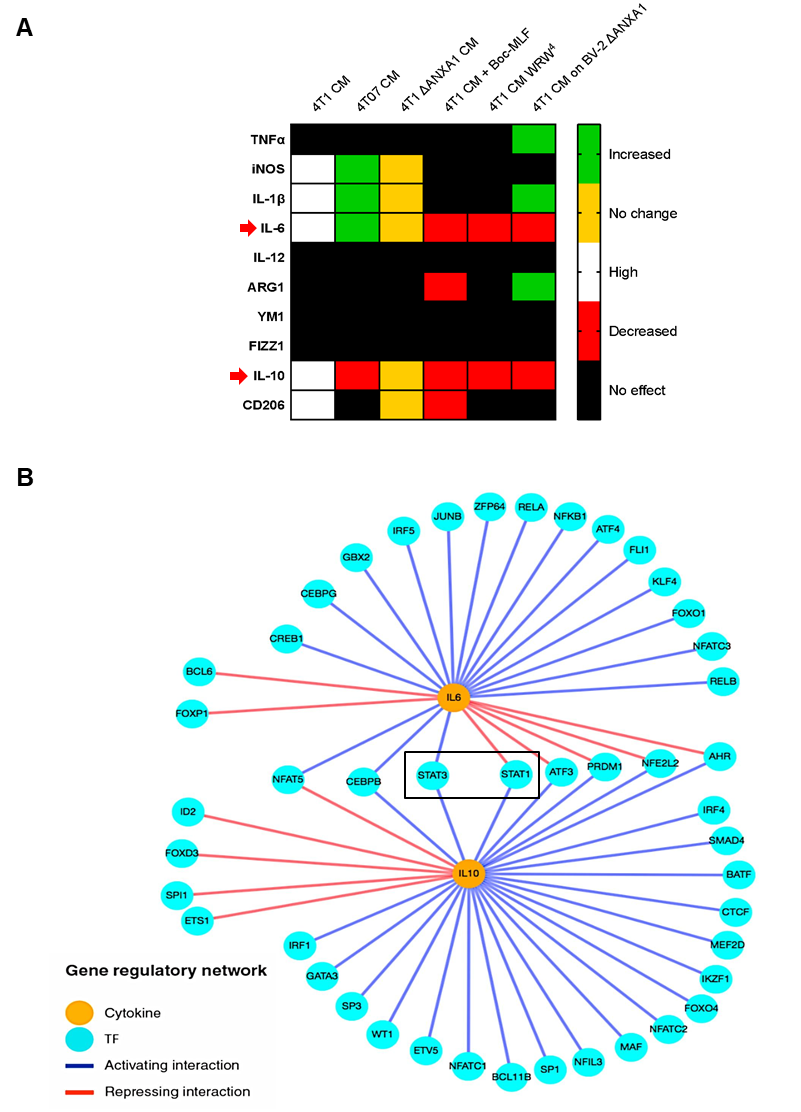


**Additional file 1: Fig. S9. Exogenous ANXA1 regulates STAT3 signalling through FPRs in microglia. (A)** Pro-inflammatory (TNFα, iNOS, IL-1β, IL-6, and IL-12) and anti-inflammatory genes (ARG1, YM1, FIZZ1, IL-10, and CD206) are presented in a table. When BV-2 microglia were treated with 4T1 CM and compared to SFM control, genes that were up-regulated are shown in white, while genes showing no change are shown in black. When BV-2 microglia treated with either 4T07 CM, 4T1 ΔANXA1 CM, 4T1 CM with 10 μM of Boc-MLF, 4T1 CM with 1 μM of WRW4 or BV-2 ΔANXA1 treated 4T1 CM, gene expressions that were increased are shown in green, gene expressions that were decreased are shown in red, while gene expressions showing no change are shown in yellow. Gene expressions that were consistently affected by the different treatments are indicated by red arrows. **(B)** Gene regulatory network from CytReg showing transcription factors that regulate IL-6 and IL-10 cytokine gene expression. Orange ovals indicate cytokine genes, cyan ovals indicate transcription factors, blue lines indicate activating interaction, red lines indicate repressing interaction, black rectangle indicate transcription factors that are shared by both IL-6 and Il-10.


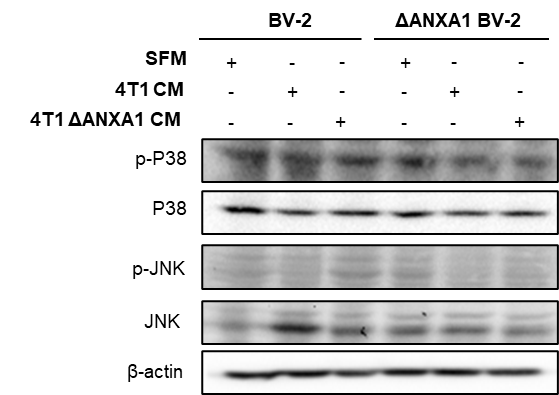


**Additional file 1: Fig. S10. Endogenous ANXA1 enhanced STAT3 activation in microglia.** Western-blot analysis showing protein expression of phosphorylated and total P38 and JNK in WT and ΔANXA1 BV-2 microglial cells treated with either SFM, 4T1 CM or 4T1 ΔANXA1 CM for 24 h. Images shown are representative of at least two independent biological replicates. Loading control: β-Actin.

**Additional file 1: Table S1 Forward and reverse primer sequences used in qRT-PCR.**

| **Gene Name (*Mu*)** | **Primer (5′→3′)** |
| --- | --- |
| *Anxa1* | F: AAGGTGTGGATGAAGCAACC |
|  | R: TGCATCAAACTGAGCTGGAG |
| *Arg1* | F: GATTATCGGAGCGCCTTTCT |
|  | R: CCACACTGACTCTTCCATTCTT |
| *CD206(Mrc1)* | F: TTGGACGGATAGATGGAGGG |
|  | R: CCAGGCAGTTGAGGAGGTTC |
| *FIZZ1(Retnla)* | F: CTGCCCTGCTGGGATGACT |
|  | R: CATCATATCAAAGCTGGGTTCTCC |
| *Fpr1* | F: CCTTGGCTTTCTTCAACAGC |
|  | R: GCCCGTTCTTTACATTGCAT |
| *Fpr2* | F: ACAGCAGTTGTGGCTTCCTT |
|  | R: CCTGGCCCATGAAAACATAG |
| *GAPDH* | F: AACTTTGGCATTGTGGAAGG |
|  | R: ACACATTGGGGGTAGGAACA |
| *IL1b* | F: CAACCAACAAGTGATATTCTCCAG |
|  | R: GATCCACACTCTCCAGCTGCA |
| *IL6* | F: GGGACTGATGCTGGTGACAA |
|  | R: TCCACGATTTCCCAGAGAACA |
| *IL10* | F: ACTGGCATGAGGATCAGCAG |
|  | R: CTCCTTGATTTCTGGGCCAT |
| *IL12 p40* | F: CACGGCAGCAGAATAAATA |
|  | R: CTTGAGGGAGAAGTAGGAATG |
| *Nos2* | F: CCGAAGCAAACATCACATTCA |
|  | R: GGTCTAAAGGCTCCGGGCT |
| *TNFa* | F: GGCAAGGATGAGCCTTTT |
|  | R: TTGGTTTGGGAGGAAAGGG |
| *YM1 (Chil3)* | F: CAAGTTGAAGGCTCAGTGGCTC |
|  | R: CAAATCATTGTGTAAAGCTCCTCTC |

F, forward; R, reverse
